# vEMINR: Ultra‐Fast Isotropic Reconstruction for Volume Electron Microscopy With Implicit Neural Representation

**DOI:** 10.1002/advs.202511922

**Published:** 2026-01-30

**Authors:** Jibin Yang, Jie Huo, Muyu Liu, Chenjie Feng, Yan Zhang, Gang Pan, Wenjia Meng, Renmin Han

**Affiliations:** ^1^ School of Software Shandong University Jinan China; ^2^ Frontiers Science Center for Nonlinear Expectations (Ministry of Education), Research Center for Mathematics and Interdisciplinary Sciences Shandong University Qingdao China; ^3^ College of Medical Information and Engineering Ningxia Medical University Yichuan China; ^4^ School of Physics Ningxia University Yinchuan China; ^5^ National Laboratory of Biomacromolecules, Institute of Biophysics Chinese Academy of Sciences Beijing China; ^6^ The State Key Lab of Brain‐Machine Intelligence Zhejiang University Hangzhou China; ^7^ Shanghai YueXin Lifescience Information Technology Company Shanghai China

**Keywords:** 3D reconstruction, deep learning, implicit neural representation, series section electron microscopy, volume electron microscopy

## Abstract

Volume electron microscopy (vEM) is a powerful technique that enables 3D visualization of biological structures at the nanometer scale. However, vEM imaging relies on sequential scanning of 2D images, and due to section thickness limitations, the axial resolution is significantly lower than the lateral resolution. In this paper, we propose the vEMINR, an ultra‐fast isotropic reconstruction method based on implicit neural representation (INR). This method enhances the reconstruction quality of vEM images by learning the true degradation patterns of low‐resolution images, and significantly accelerates the reconstruction process by utilizing the efficient parameterization and a continuous function representation of INR. In experiments on 11 public datasets, vEMINR outperforms mainstream methods with over tenfold faster reconstruction and higher accuracy. vEMINR substantially improved the accuracy of organelle and neuron reconstruction from vEM. Overall, the excellent reconstruction time efficiency of vEMINR enables high‐throughput processing of terabyte‐scale vEM datasets while maintaining reconstruction accuracy. We believe that it will play a significant role in large‐scale vEM image reconstruction and related research fields.

## Graphical abstract

vEMINR is an ultra‐fast isotropic reconstruction method for vEM based on implicit neural representation, achieving over tenfold faster reconstruction and higher accuracy on 11 datasets, showing strong potential for large‐scale vEM data processing.

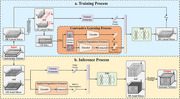

## 1 Introduction

Volume electron microscopy (vEM) is a powerful technique that enables 3D visualization of biological structures at nanometer resolution [1, 2]. Recent rapid developments have expanded the physical scale of vEM imaging from cubic micrometers to hundreds of micrometers [3, 4], and further promoted its widespread application in life sciences [5, 6], medicine [7, 8], and clinical diagnostics [9, 10, 11]. For example, vEM technology is widely used in the brain connectomics research [12, 13, 14, 15, 16, 17] and cancer research [18, 19, 20]. vEM methods are typically based on serial section imaging of biological sample sections [21]. First of all, the sample is continuously sectioned along the z‐axis. Then, each section is stained and digitally imaged. Finally, these 2D images are stitched and 3D registered to reconstruct the complete 3D biological structure. A medium‐sized sample can produce tens to hundreds of terabytes of data, posing a substantial challenge to processing efficiency in vEM studies.

The sections of biological samples used for vEM imaging contain only the integrated information of each thickness of the section, so the thickness of the section determines the axial voxel size [22]. Currently, most diamond knife cutting methods can only prepare sample sections with a thickness of tens of nanometers, which is >10 times the lateral voxel size. This causes a mismatch between the lateral and axial resolutions, resulting in anisotropic imaging of the vEM. Focused Ion Beam Scanning Electron Microscopy (FIB‐SEM) techniques [23, 24], which utilize ion beam milling to produce ultrathin slices are slower, have a limited field of view, and are not as easily available as conventional EM. Such anisotropic volumes further constrain the accuracy of subsequent analysis, thus, achieving high‐quality isotropic reconstruction becomes a crucial task in the vEM data process.

Interpolation methods have long served as baseline approaches for vEM isotropic reconstruction, traditionally applied to mitigate anisotropy resulting from the loss of axial information [25, 26, 27]. These methods estimate the missing slice values using the surrounding known textures under the assumption of biological data continuity and smoothness. However, simple interpolation often fails to capture the complexity of biological structures, resulting in noticeable blurriness and stair‐step artifacts. Recently, deep learning methods have been introduced to recover axial information in vEM. Supervised learning methods [28] were introduced to solve the isotropic reconstruction problem in volume electron microscopy. However, supervised learning's dependence on ground truth data limits its applicability in real‐world scenarios. Later, more effort was put into self‐supervised learning methods. Meanwhile, similar tasks have also been studied in other microscopic imaging fields, such as the restoration of anisotropic point spread functions in 3D fluorescence super‐resolution imaging [29], the super‐resolution problem of slice imaging in scanning microscopy [30], and the correction of anisotropic deformations in cryo‐electron tomography [31]. In vEM isotropic reconstruction field, self‐supervised learning methods based on diffusion models [32] have demonstrated more powerful reconstruction capabilities and overcome the reliance on ground truth data. Although this method demonstrates improved performance over the baseline, its high computational complexity and slow processing speed limit its applicability in isotropic reconstruction tasks. Given that vEM technologies generate datasets ranging from tens to hundreds of terabytes, this creates major computational challenges, making it a key issue in this field to significantly improve the reconstruction time efficiency while maintaining reconstruction accuracy.

In this work, we propose the vEMINR, an ultra‐fast self‐supervised isotropic reconstruction method based on implicit neural representation. Similar to unsupervised methods, vEMINR does not rely on real 3D isotropic ground truth during training; however, unlike them, it leverages high‐resolution XY slices from anisotropic volumes as supervision to guide the reconstruction process. Our approach restores missing axial information by learning implicit neural representations (INR) of real vEM textures. vEMINR employs a multilayer perceptron (MLP) to learn a continuous representation of high‐resolution signals, enabling precise decoding of low‐resolution (LR) axial features into the high‐resolution (HR) texture space at arbitrary spatial coordinates. This decoding process does not require complex image generation steps, significantly reducing memory consumption and computational resource overhead. Furthermore, vEMINR adopts a self‐supervised contrastive learning approach, learning the degradation function of real LR images by minimizing intra‐image differences and maximizing inter‐image differences, thus addressing the mismatch between axial and lateral degradation information. We validate vEMINR on seven simulated datasets and three real datasets, containing various cell types and biological structures. The results show that, compared to the state‐of‐the‐art diffusion model‐based methods, vEMINR further improves isotropic reconstruction quality while significantly enhancing time efficiency (by 17 times). Additionally, vEMINR demonstrates robustness in domain transfer experiments, effectively handling data from different sources and types, and performing reconstruction tasks at arbitrary scales (including fractional ones). In conclusion, vEMINR provides a fast and accurate approach for vEM isotropic reconstruction, further advancing the understanding of complex biological structures and the development of related fields.

## 2 Results

### 2.1 Workflow of vEMINR Procedure

**FIGURE 1:**
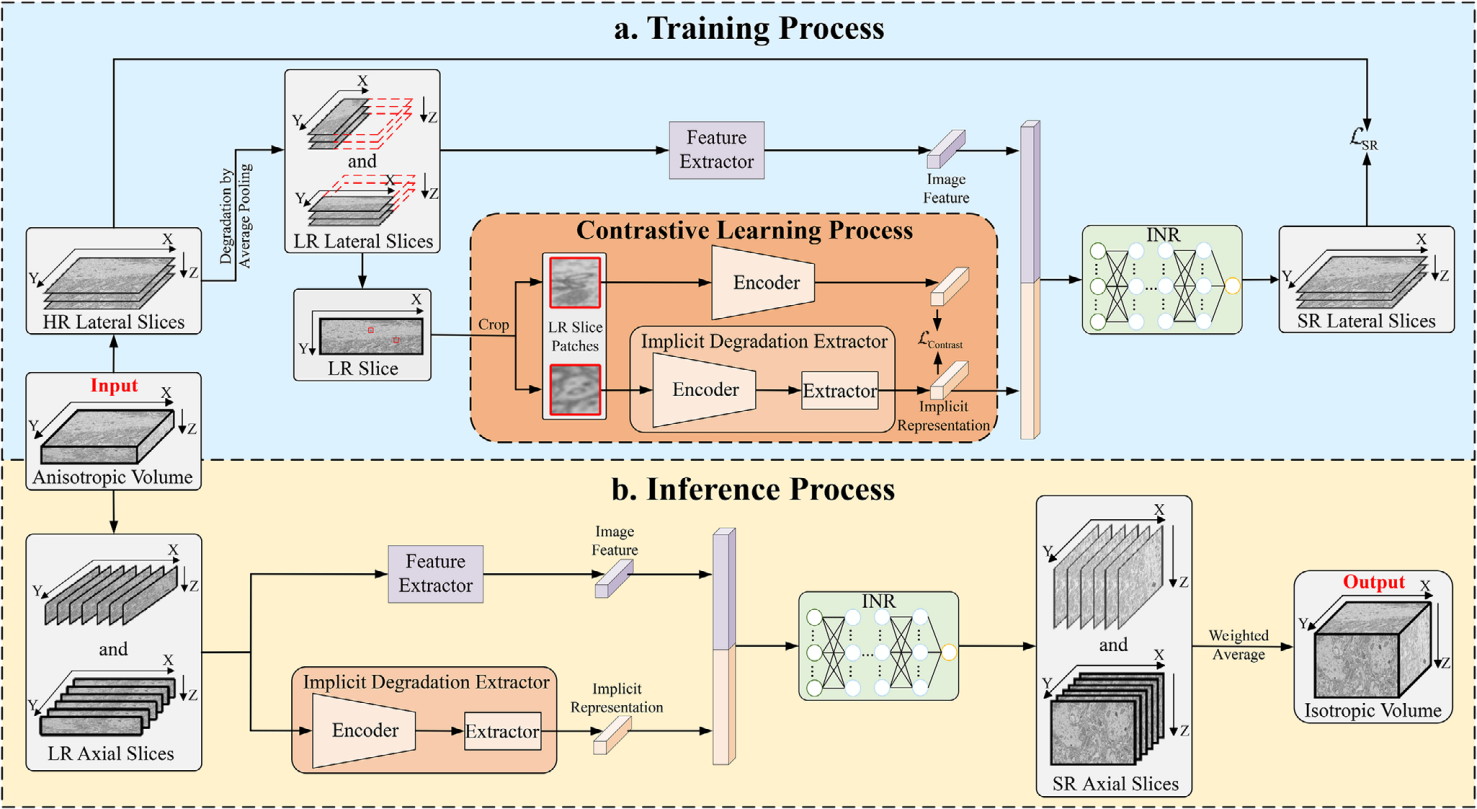
Workflow of vEMINR. (a) Training process of vEMINR. During the training process, the high‐resolution (HR) transverse (XY) slices of the anisotropic volume are manually degraded to generate low‐resolution (LR) input slices. These LR slices pass through three modules—feature extractor, degradation extractor, and implicit neural representation (INR)—to upsample and generate super‐resolution (SR) images. The degradation extractor is trained using a contrastive learning method, while the feature extractor and INR module are optimized by comparing the SR images with the HR images. (b) Inference process of vEMINR. During the inference process, vEMINR separately reconstructs two LR axial slices (XZ and YZ) to obtain two reconstructed volumes, which are then combined through weighted averaging to produce the final isotropic volume.

Figure 1 illustrates the architecture of vEMINR, which consists of three main modules: a feature extractor, an implicit degradation extractor, and an implicit neural representation module. It also outlines the overall workflow for model training and inference processes.

Since isotropic vEM volumes are generally difficult to obtain, there is often a lack of high‐resolution Z‐axis slices that can guide reconstruction. In contrast, planar XY slices are less affected by factors such as sample thickness and therefore usually have higher resolution. Based on this characteristic, we treat the input anisotropic volume as a stack of high‐resolution (HR) XY slices along the Z‐axis. To construct training data pairs, we apply average pooling to simulate the degradation of these HR slices along the X/Y axes, thereby generating the corresponding low‐resolution (LR) slices. The simulated LR slices are processed by a feature extractor and an implicit neural representation (INR) module [33, 34], which performs super‐resolution (SR) along the simulated degradation axis (X or Y). To optimize the network, we compute the L1 loss between each SR slice and its corresponding HR XY slice (Figure 1a). Notably, to enhance isotropic reconstruction, we develop an implicit degradation extractor that captures global degradation patterns rather than relying solely on local information from low‐resolution slices (contrastive learning process in Figure 1a). This global understanding of degradation helps the reconstruction module compensate for depth inconsistencies between the XY and Z directions, thereby alleviating the limitations of self‐supervised methods [29, 30, 35] which overlook that the pixels in XY slices are deeper than truly isotropic pixels.

While the proposed vEMINR method is applied to 3D vEM data, it is essentially a 2D‐architecture self‐supervised learning framework. Specifically, it performs super‐resolution reconstruction on low‐resolution axial slices (see the inference process in Figure 1b) and then reconstructs the resulting images into 3D isotropic volumes (see Equations (29) and (30)), following a widely adopted a twostage strategy in this field [28, 32, 36, 37, 38].

### 2.2 Isotropic Reconstruction

#### 2.2.1 Isotropic Reconstruction on Simulated Data

**FIGURE 2:**
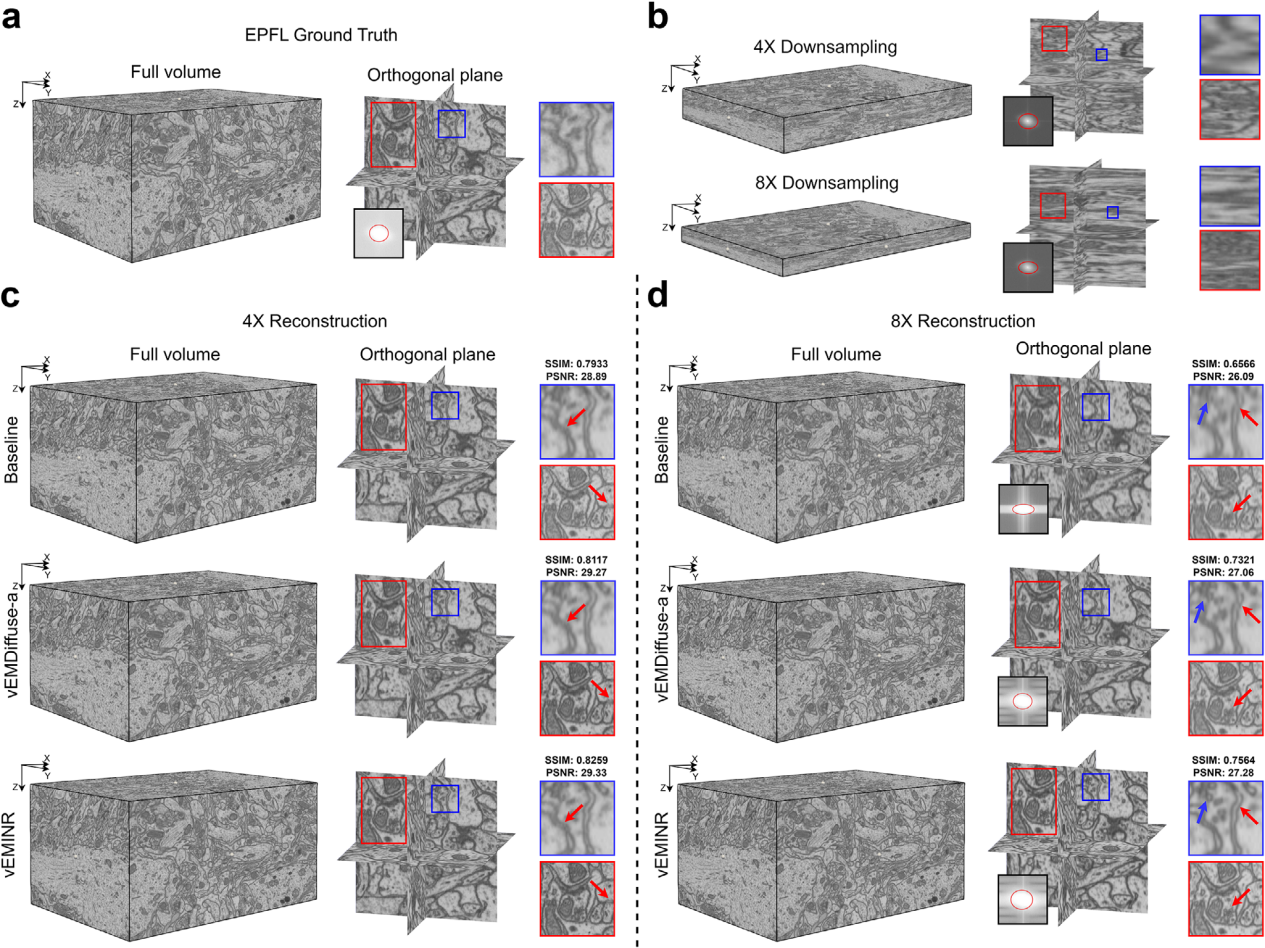
Our method performs isotropic reconstruction tasks well on simulated dataset. (a) Isotropic EPFL dataset used as ground truth. (b) Simulated low‐resolution datasets after downsampling with average pooling. 4× downsampling (top), 8× downsampling (bottom). (c) Full volume (left) and orthogonal plane (right) of reconstruction results of various methods on the simulated dataset EPFL with 4× anisotropy. Blue boxes: bilayer membranes, red boxes: mitochondrial ridge. (d) Full volume (left) and orthogonal plane (right) of the reconstruction results of various methods on the 8× anisotropic simulated data set EPFL. The lower left corner of the orthogonal plane is the Fourier power spectrum of the XZ plane.

To validate the reconstruction capability of vEMINR, we perform isotropic reconstruction on seven simulated datasets with 4× and 8× anisotropy. The orthogonal views of the reconstruction results show that vEMINR consistently restores the most detailed structural features in all cases (Figure 2; Figures S1 and S2). Taking the EPFL simulation dataset as an example. We first perform 4× and 8× average pooling downsampling along the Z‐axis of the isotropic volume (Figure 2a). As shown in Figure 2b, the simulated low‐resolution data generated through downsampling exhibits a loss of structural information. However, in the lower 4× reconstruction, vEMINR restores the finest details of textures (Figure 2c). This finding becomes even more pronounced in the higher 8× reconstruction. Compared to other methods, vEMINR effectively recovers the most isotropic information in both the Fourier space and image space (Figure 2d). The Fourier power spectrum of the XZ plane indicates that the Fourier space information retrieved by vEMINR is consistent with the ground truth, whereas other methods suffer from varying degrees of anisotropic resolution. In the 8× reconstruction results on the EPFL simulated dataset (Figure 2d), as indicated by the red arrows, vEMINR achieves a more continuous reconstruction of the bilayer membrane structures and produces clearer vesicle structures compared with vEMDiffuse. vEMDiffuse while recovering axial information, still introduce a certain degree of blurring. The reconstruction results produced by cubic show significant blurring and artifacts as the scale increases. Notably, vEMINR generates some vesicle structures during reconstruction that are not observed in ground truth (indicated by the blue arrows in Figure 2d). These potential hallucinations in the INR‐based vEMINR method may stem from its single deterministic mapping that predicts pixel intensities at arbitrary spatial coordinates. Such a deterministic formulation forces the network to output a single, continuous estimation even in regions with incomplete or ambiguous information, causing it to over‐smooth spatial details and potentially generate fictitious or blurred structures. In contrast, vEMDiffuse‐a samples multiple plausible solutions, allowing for the preservation of structural uncertainty and reducing the risk of producing such hallucinations. Despite the deterministic design of vEMINR, our method offers substantial advantages: it enables stable and efficient inference using INR, significantly improving computational efficiency while ensuring superior reconstruction quality.

We evaluate the reconstruction quality using 3D and orthoplane (XY, XZ, YZ) PSNR/SSIM/LPIPS metrics. Specifically, the 3D metrics are computed between the reconstructed and ground‐truth 3D volumes, whereas the orthoplane metrics are obtained by averaging the PSNR/SSIM/LPIPS values calculated for all reconstructed 2D orthogonal slices against their corresponding 2D ground‐truth slices. (Table 1, Table S1 and Figures S3–S6). As shown, vEMINR consistently outperforms other methods across all metrics. As shown in Table 1, in the 4× reconstruction experiment, vEMINR outperformed all comparison methods (Baseline and vEMDiffuse‐a) in reconstruction accuracy, achieving the highest SSIM and PSNR values and the lowest LPIPS scores. For example, the vEMINR method achieved a 3D SSIM of 0.8259, a 3D PSNR of 29.33, and LPIPS values of 0.1224, 0.4241, and 0.4265 for the XY, XZ, and YZ planes, respectively. In the 8× reconstruction experiment, vEMINR consistently maintained superior reconstruction accuracy compared with both the Baseline and vEMDiffuse‐a methods. This result is consistent with the observation that vEMINR restores the most detailed structural features (Figure 2). Meanwhile, the LPIPS metric across all orthogonal planes further indicates that vEMINR not only reconstructs better structural details and smaller pixel differences, but also ensures higher visual similarity.

**TABLE 1:** Validation metrics for different methods on EPFL dataset.

Scale	Method\Metric	SSIM ↑				PSNR ↑				LPIPS ↓		
		3D	XY	XZ	YZ	3D	XY	XZ	YZ	XY	XZ	YZ
4×	Baseline	0.7933	0.7696	0.7484	0.7436	28.89	28.93	28.89	28.89	0.2415	0.5248	0.5261
	vEMDiffuse‐a	0.8117	0.7921	0.7786	0.7618	29.27	29.30	29.26	29.29	0.2289	0.5067	0.5065
	vEMINR	**0.8259**	**0.7981**	**0.7830**	**0.7782**	**29.33**	**29.36**	**29.32**	**29.35**	**0.1224**	**0.4241**	**0.4265**
8×	Baseline	0.6566	0.6566	0.6078	0.6000	26.09	26.21	26.09	26.09	0.3650	0.6117	0.6135
	vEMDiffuse‐a	0.7321	0.7319	0.7138	0.7009	27.06	26.94	26.86	26.85	0.3321	0.5839	0.5823
	vEMINR	**0.7564**	**0.7541**	**0.7346**	**0.7237**	**27.28**	**27.21**	**27.12**	**27.11**	**0.2352**	**0.5132**	**0.5178**

#### 2.2.2 Isotropic Reconstruction on Real Data

**FIGURE 3:**
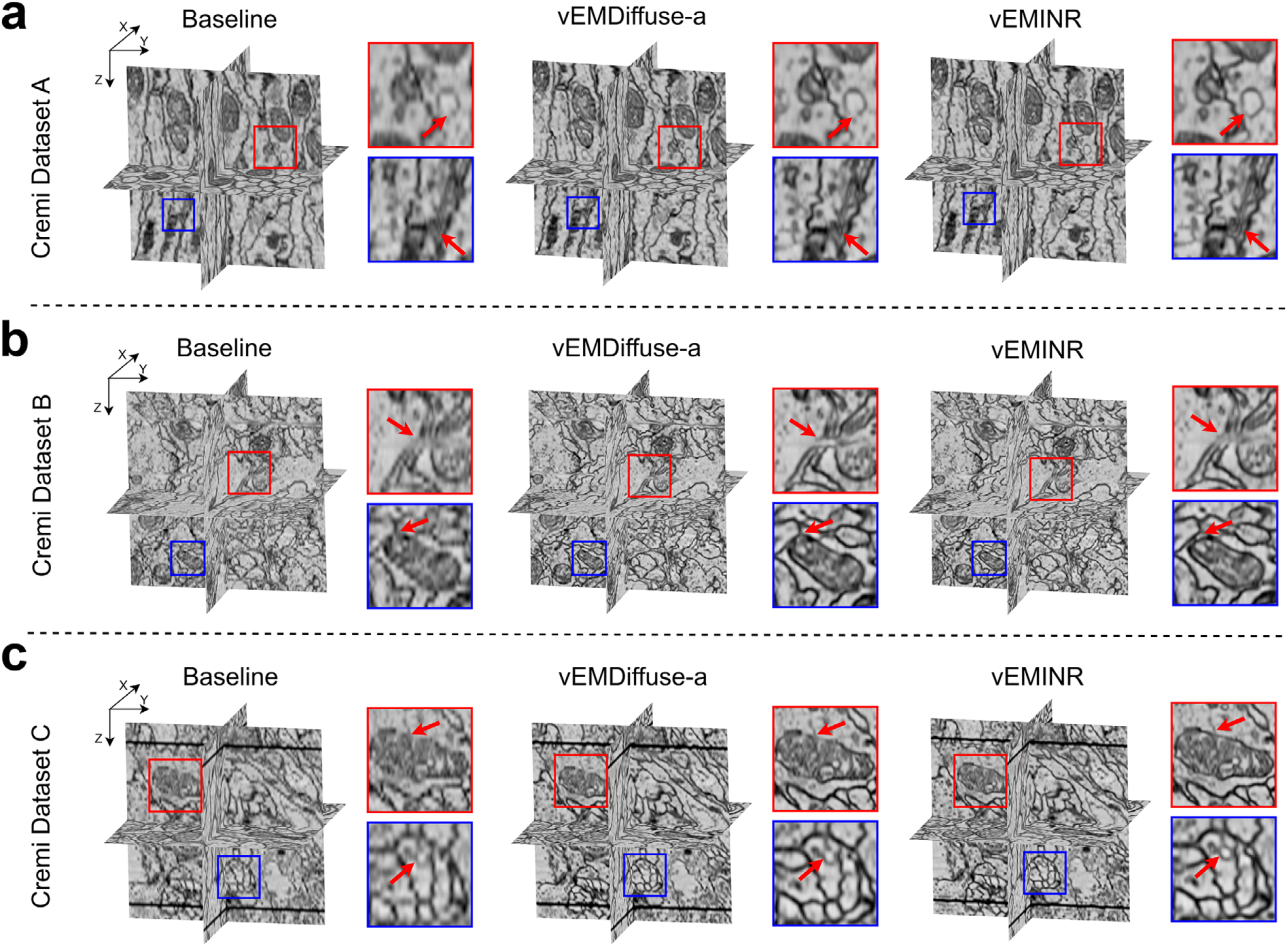
Our method performs isotropic reconstruction tasks well on real dataset. (a) Reconstruction results of various methods on the Cremi Dataset A (anisotropic factor 10). (b) Reconstruction results of various methods on Cremi Dataset B (anisotropic factor 10). (c) Reconstruction results of various methods on Cremi Dataset C (anisotropic factor 10).

To further verify the effectiveness of vEMINR and its applicability across various vEM modalities, we apply it to three real datasets—Cremi dataset A, Cremi dataset B, and Cremi dataset C—each collected using ssTEM with different morphological structures and an anisotropy factor of 10 (Figure 3). As shown in the reconstruction results, vEMINR reconstructs the most detailed structural information. In contrast, although vEMDiffuse‐a can recover certain axial information, it still exhibits blurriness in some membrane regions, as indicated by the red arrows in Figure 3. The reconstruction results produced by cubic are the worst, with significant artifacts and blurriness in the axial direction, almost failing to recover axial information. These findings indicate that vEMINR generates isotropic 3D data from non‐isotropic inputs that looks plausible and improves standard pixel based metrics and frequency characteristics over established techniques.

### 2.3 3D Segmentation of Isotropic Reconstruction Results

**FIGURE 4:**
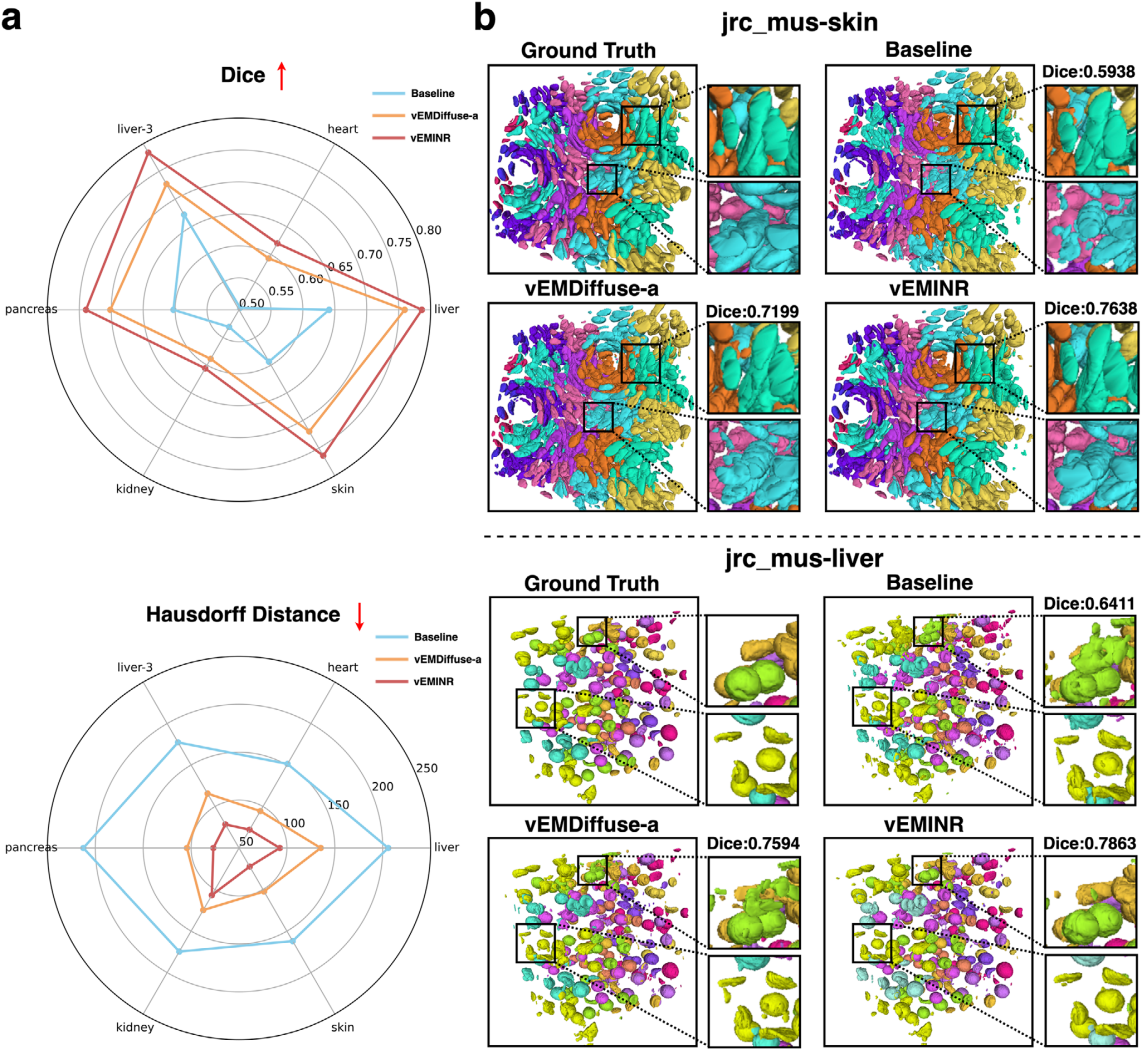
Our methods show excellent performance in downstream 3D segmentation tasks. (a) Comparison of 3D segmentation accuracy (Dice and Hausdorff Distance) across six different simulated datasets for different methods. (b) Visualization of 3D segmentation results on the jrc_mus‐skin and jrc_mus‐liver datasets (with an anisotropy factor of 8).

Volume Electron Microscopy (vEM) 3D segmentation is a key downstream task in isotropic reconstruction, widely applied in tissue recognition [39] and cell segmentation [1]. This process significantly enhances biologists' understanding of cellular structures [40]. As six real isotropic FIB‐SEM from the Open Organelle platform contain reliable segmentation annotations, we conduct 3D segmentation on their 8× reconstruction results to evaluate the effectiveness of vEMINR in downstream tasks. To this end, we train an advanced 3D segmentation model [41] on each simulated dataset. We quantify the segmentation results using the Dice score and Hausdorff distance (Table S2) and present them visually in radar charts (Figure 4a). As shown, our method consistently achieves the highest Dice scores and the smallest Hausdorff distances across all datasets, demonstrating the positive impact of vEMINR's excellent reconstruction capability on downstream tasks.

**FIGURE 5:**
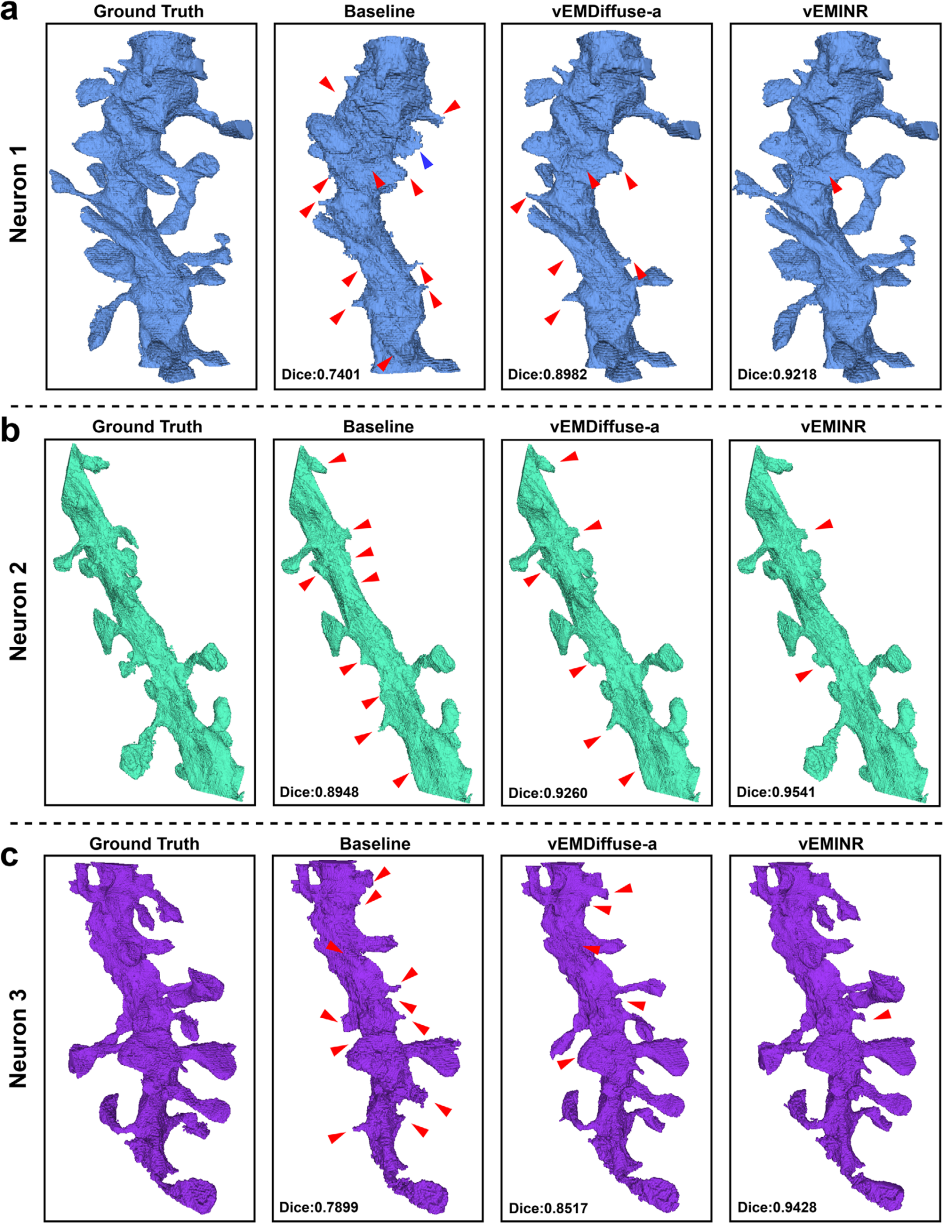
Our method provides excellent technical support for research in the field of connectomics. (a) The 3D visualization result of the first neuron segmented from the 8× reconstructed mouse brain dataset EPFL(The red triangles represent missing segmentation regions, the blue triangles indicate erroneously merged regions, and the Dice metric in the bottom left corner represents the colocalization analysis result). (b) The 3D visualization result of the second neuron. (c) The 3D visualization result of the third neuron.

Figure 4b visualizes the 3D segmentation results on the jrc_mus‐skin and jrc_mus‐liver datasets (segmentation results on other simulated datasets are visualized in Figure S7). As shown, the segmentation results of the vEMINR reconstructed volumes are closer to the ground truth compared to other methods. Additionally, we performed more advanced neuronal segmentation experiments on the 8× reconstruction results of the EPFL mouse brain dataset (Figure 5). The results indicate that our method consistently retains the finest segmentation details and outperforms all compared methods in terms of Dice score. This further demonstrates that vEMINR's precise structural reconstruction significantly improves 3D segmentation quality in downstream tasks, providing stronger support for detailed analysis of internal biological structures.

### 2.4 Neuron Segmentation Results in Real Connectomics Datasets

**FIGURE 6:**
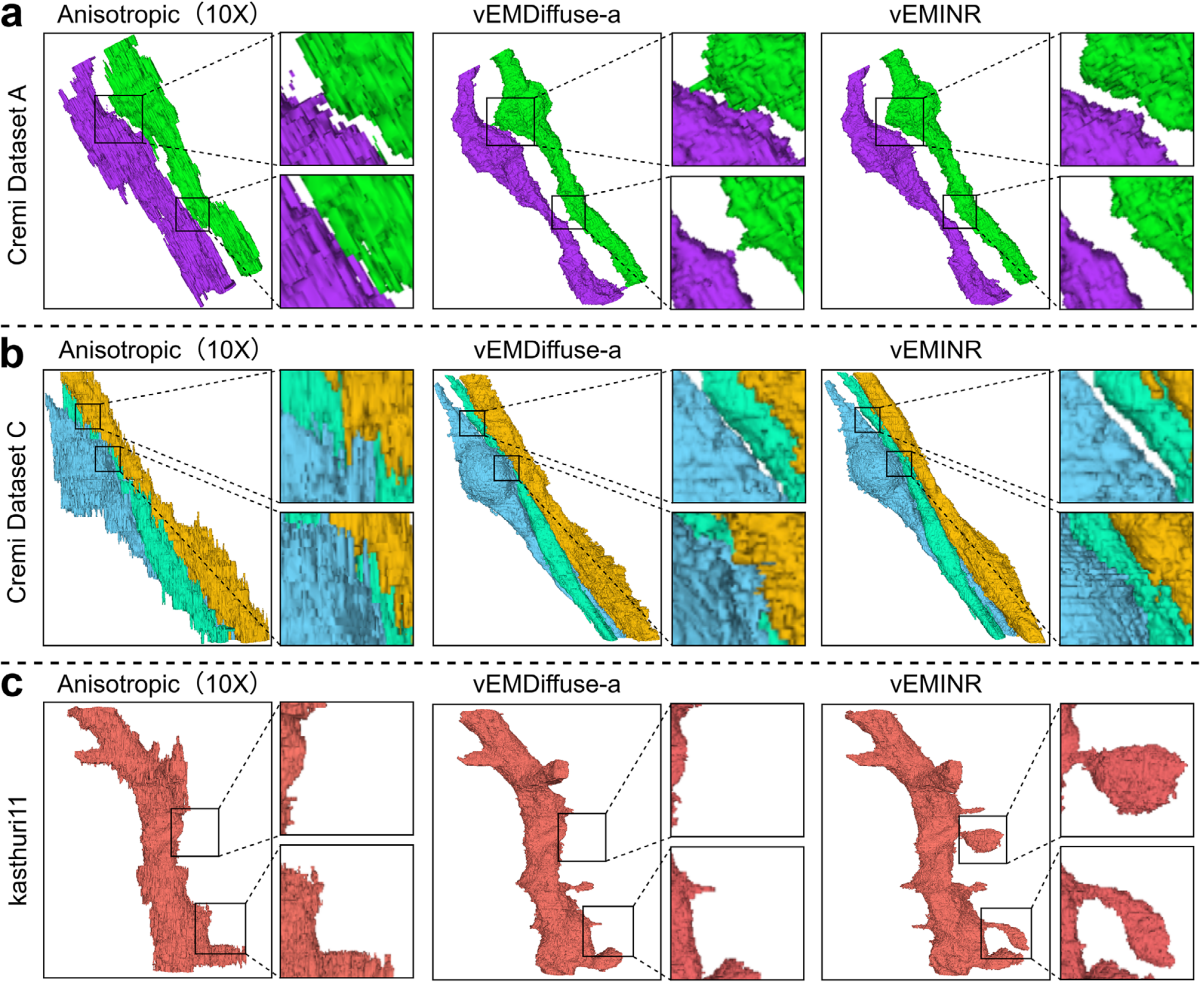
Our method can facilitate connectomics research on real datasets. (a) Perform 10× isotropic reconstruction on the Cremi Dataset A and visualize the 3D neuron segmentation results before and after reconstruction. (b) Perform 10× isotropic reconstruction on the Cremi Dataset C and visualize the 3D neuron segmentation results before and after reconstruction. (c) Perform 10× isotropic reconstruction on the Kasthuri11 dataset and visualize the 3D neuron segmentation results before and after reconstruction.

Connectomics has recently emerged as a prominent research area, with isotropic reconstruction serving as a fundamental enabling technology. In this section, we present the neuron segmentation results based on the reconstructions obtained from deep learning methods, to qualitatively validate vEMINR‘s effectiveness in practical connectomics scenarios. The results in Figure 6 indicate that, compared with vEMDiffuse‐a, vEMINR achieves more accurate restoration of synaptic connections between neurons and a more complete reconstruction of neuronal structures, thereby providing a stronger foundation for connectomics analysis. Since ground truth is not available for real datasets, Dice scores, which quantify the overlap between predictions and ground truth, are not reported in Figure 6.

Specifically, Figure 6a presents the segmentation results of two adjacent axonal structures obtained from a 10× reconstruction on the CremiDataset A, demonstrating that vEMINR accurately restores the spatial topology of axons, while vEMDiffuse‐a introduces erroneous neuronal connections due to blurred boundaries. To further validate this, we performed a 10× reconstruction on the Cremi Dataset C and extracted three interconnected neuronal axons (Figure 6b). The experimental results indicate that vEMINR clearly preserves the axon‐soma connection patterns, while vEMDiffuse‐a introduces incorrect neuronal connections due to reconstruction errors. Furthermore, in the Kasthuri11 dataset, we extracted a complete basal dendrite along with its dendritic spines (Figure 6c). The results show that vEMINR effectively reconstructs the detailed dendritic spine structures, whereas vEMDiffuse‐a fails to maintain connectivity in narrow regions, leading to structural loss. Figure 6 demonstrates that vEMINR achieves accurate isotropic reconstructions on real connectomics datasets, facilitating improved neural circuit construction and serving as a powerful tool for connectomics studies.

### 2.5 Transfer Ability and Robustness Analysis

In practical applications, vEM isotropic reconstruction often encounters the limitation that models trained on one dataset are difficult to apply to other datasets or scale factors, which can significantly affect the model's performance. To validate the transferability of our method, we compare the proposed vEMINR with vEMDiffuse‐a through cross‐dataset reconstruction experiments, i.e., transferring from the Cremi Dataset C to the EPFL dataset and vice versa. Specifically, the transfer from the Cremi Dataset C to the EPFL dataset corresponds to training on the Cremi dataset and evaluating on the FIB‐SEM dataset to assess transferability, and vice versa. It is worth noting that the Cremi dataset exhibits an anisotropy factor of 10, determined by its real‐data acquisition process, whereas the simulated EPFL dataset, acquired by FIB‐SEM, has a different anisotropy factor of 8, which further helps to evaluate transferability.

**FIGURE 7:**
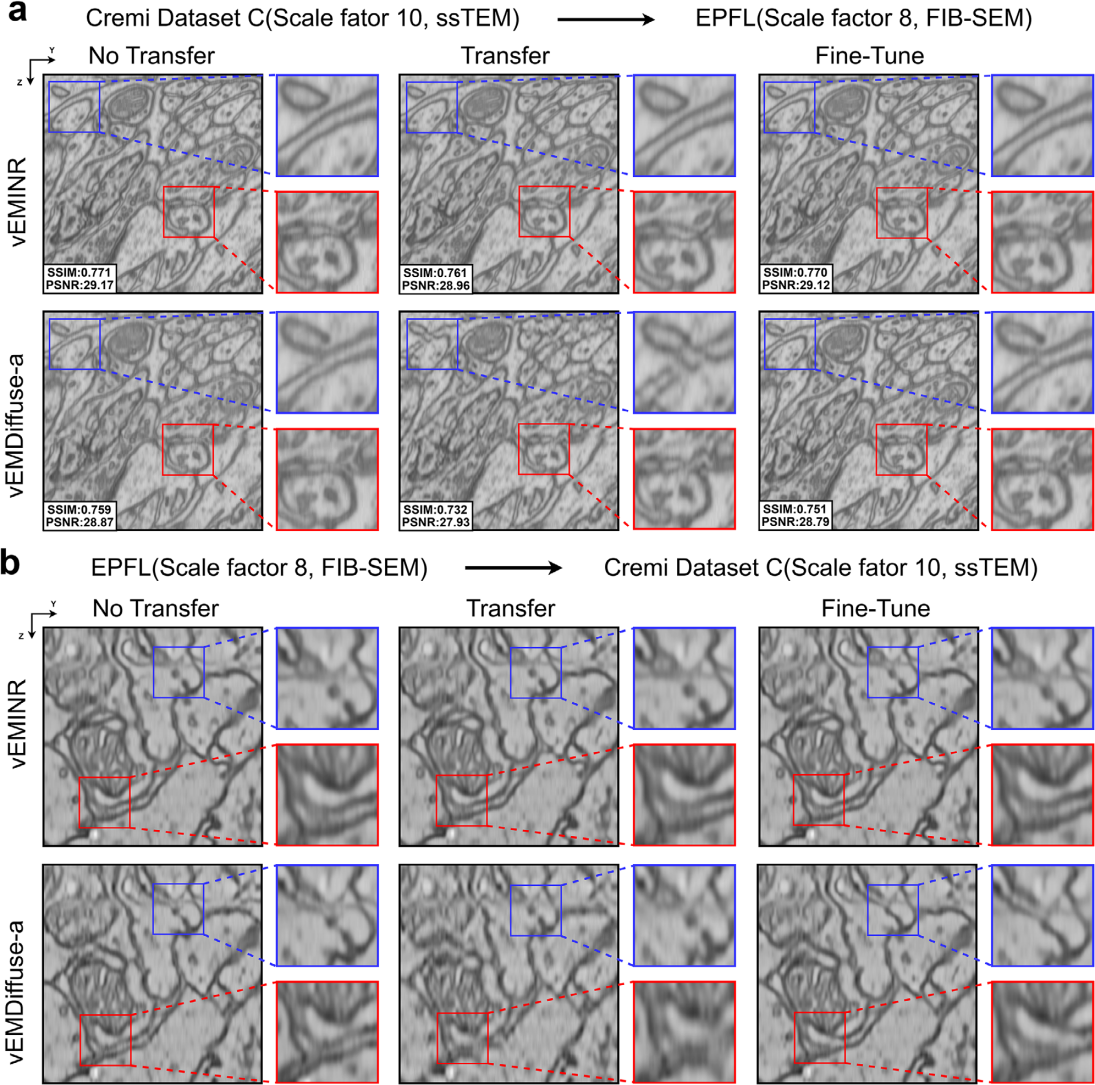
Our method has strong transfer learning capabilities. (a) Results of the EPFL dataset (simulated 8× downsampled) is reconstructed using the vEMINR and vEMDiffuse‐a, which were pre‐trained on Cremi‐Dataset C (10× anisotropy). (b) Results of the Cremi‐Dataset C (10× anisotropy) is reconstructed using the and vEMDiffuse‐a, which was pre‐trained on EPFL dataset (simulated 8× downsampled).

The experimental results are shown in Figure 7, where Figure 7a illustrates the transfer from the CREMI Dataset C to the EPFL dataset(Cremi Dataset C → EPFL), and Figure 7b shows the reverse transfer(EPFL → Cremi Dataset C). We report results under three settings: “No Transfer” where the model is trained and tested on the target dataset; “Transfer” where the model is trained on a source dataset and tested on a target dataset; and “Fine‐Tune” where the model is pretrained on a source dataset and lightly fine‐tuned on the target dataset before testing. As shown in Figure 7a, when transferring from the Cremi Dataset C to the EPFL dataset, vEMINR maintains high reconstruction accuracy (SSIM = 0.761; PSNR = 28.96) under the “Transfer” setting, particularly preserving fine structural details such as membrane boundaries and intracellular textures. Compared with the “No Transfer” setting (SSIM = 0.771; PSNR = 29.17), its performance only shows a slight decline. In contrast, vEMDiffuse‐a exhibits obvious degradation during transfer, with PSNR and SSIM decreasing from 28.87 / 0.759 in “No Transfer” to 27.93 / 0.732 in “Transfer”, showing ineffective recovery of membrane details and producing blurred or incomplete boundaries. In the reverse transfer (EPFL → CREMI Dataset C), vEMINR demonstrates robust cross‐domain transferability, as shown in Figure 7b. Under the “Transfer” setting, it accurately reconstructs membrane boundaries and intracellular textures, with only a minor performance drop that can be quickly recovered through light fine‐tuning. In contrast, vEMDiffuse‐a exhibits blurred boundaries, missing details, and discontinuous textures. Overall, these results suggest that vEMINR has good generalization and cross‐domain transfer capabilities, achieving high‐quality isotropic reconstruction with direct transfer or light fine‐tuning, and indicating its potential for practical applications.

### 2.6 Arbitrary Scale Isotropic Reconstruction

In vEM isotropic reconstruction, the anisotropy factors are typically of arbitrary scales (both integer and non‐integer), which makes the ability to perform reconstruction at arbitrary scales particularly important for practical applications. The proposed vEMINR method for vEM isotropic reconstruction is built upon the framework of Implicit Neural Representation (INR). In this framework, spatial coordinates are fed into a neural network that predicts the corresponding pixel intensities, thereby achieving isotropic reconstruction. Since the input coordinates can take any real‐valued position rather than being limited to integer grids, vEMINR enables isotropic reconstruction at arbitrary scales, distinguishing it from existing methods, such as vEMDiffuse, which generates slices only at integer multiples and is therefore limited to integer‐scale reconstruction. As demonstrated in the previous experiments, our method has been validated on data with integer‐multiple anisotropy factors. In this section, we further conduct experiments on data with non‐integer anisotropy factors to evaluate the reconstruction performance of vEMINR.

**FIGURE 8:**
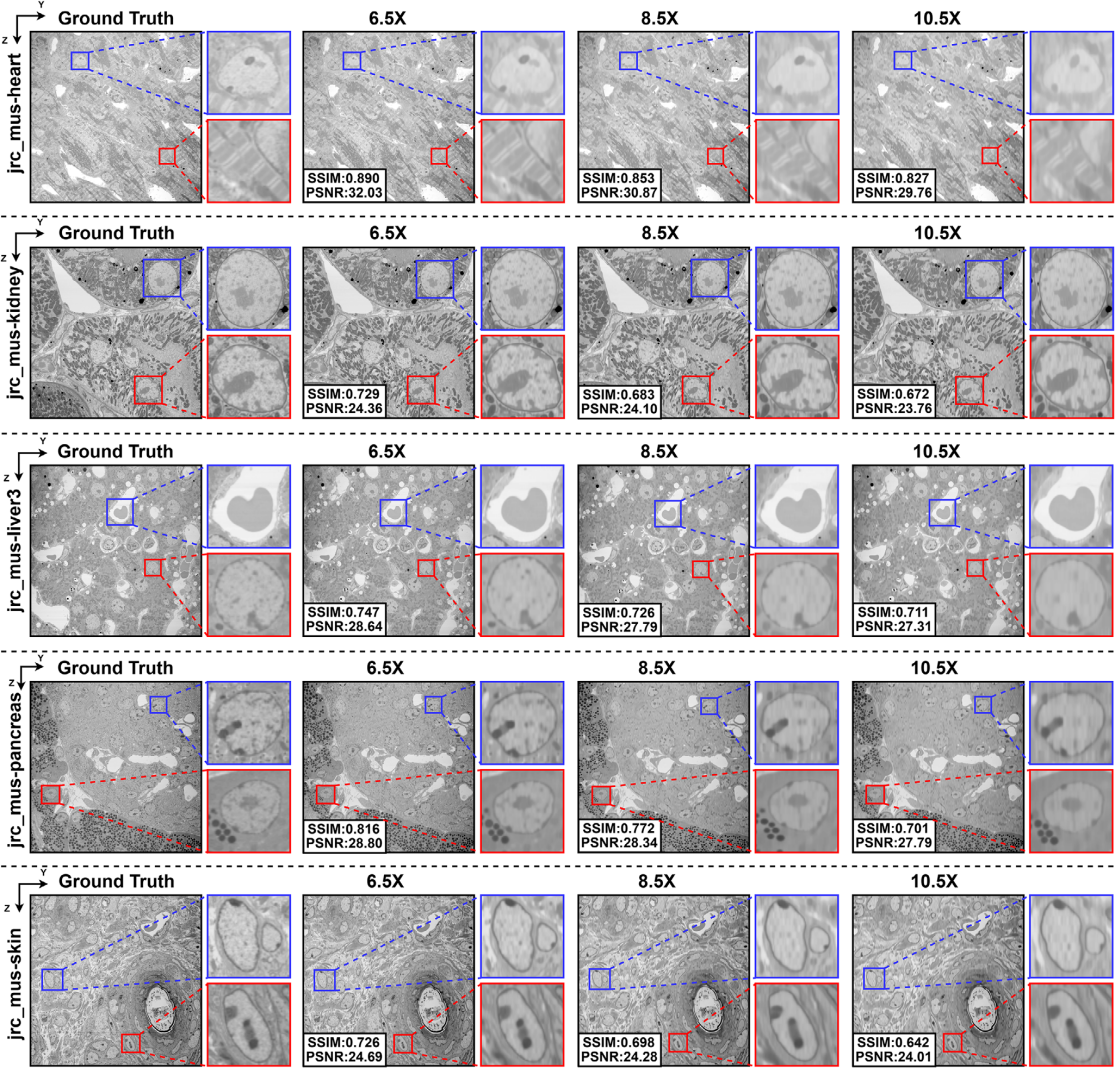
Our method can achieve isotropic reconstruction at arbitrary scale. Reconstruction results of vEMINR on five simulated datasets (jrc_mus‐heart, jrc_mus‐kidney, jrc_mus‐liver3, jrc_mus‐pancreas, and jrc_mus‐skin) with anisotropy factors of 6.5, 8.5, and 10.5.

To experimentally validate the capability of our method for isotropic reconstruction at arbitrary resolutions, we trained and tested vEMINR on the jrc_mus‐heart, jrc_mus‐kidney, jrc_mus‐liver3, jrc_mus‐pancreas, and jrc_mus‐skin datasets. The results are presented in Figure 8, where each row corresponds to one dataset and the four columns represent the ground truth and reconstructions at anisotropy factors of 6.5, 8.5, and 10.5. As shown in Figure 8, vEMINR successfully recovers rich axial structural information that closely matches the ground truth under the “6.5×” and “8.5×” settings. For example, in the jrc_mus‐kidney and jrc_mus‐skin datasets, vEMINR accurately reconstructs continuous membrane boundaries; moreover, in the jrc_mus‐pancreas dataset, it also recovers well‐defined secretory granule structures. Some detail loss is observed at the “10.5×” setting relative to the ground truth, mainly due to the higher anisotropy factor. These results demonstrate that our method can robustly perform isotropic reconstruction across arbitrary scales.

### 2.7 Time Efficiency Analysis

In isotropic reconstruction tasks, time efficiency is crucial for handling large‐scale vEM data on the order of hundreds of terabytes. This reconstruction process is typically computationally intensive and time‐consuming. Our method significantly reduces reconstruction time by introducing implicit neural representations. Specifically, this method encoder and processes data in a more efficient neural network structure, rather than relying on traditional high‐dimensional dense data representations, thus significantly reducing computational complexity and improving time efficiency.

To verify the time efficiency advantage of vEMINR, we also test the reconstruction time overhead of vEMDiffuse‐a under the same conditions (Table 2). The results demonstrate that vEMINR achieves over a 10‐fold speedup compared to vEMDiffuse‐a, e.g., on the Cremi Dataset A, where vEMDiffuse‐a required 535 min, while vEMINR completed the reconstruction in only 36 min. Furthermore, during the experiments, we observe that due to the computation‐intensive diffusion model employed by vEMDiffuse‐a, each input image must be cropped into 256×256 patches, which are then stitched together after inference to obtain the final output image. In contrast, vEMINR does not require this post‐processing step. This further demonstrates that, within the same time frame, vEMINR can effectively reconstruct larger‐scale vEM data. We anticipate that this significant performance improvement makes a substantial contribution to research and applications in related fields.

**TABLE 2:** Comparison of processing times (in min) across different methods and datasets.

Methods	Scale	EPFL	jrc_mus‐heart	jrc_mus‐kidney	jrc_mus‐liver	jrc_mus‐liver3	jrc_mus‐pancreas	jrc_mus‐skin	Cremi A	Cremi B	Cremi C	kasthuri11
vEMDiffuse‐a	4×	1517	568	1562	177	431	379	435	—	—	—	—
	8×	1639	621	1602	206	462	413	478	—	—	—	—
	10×	—	—	—	—	—	—	—	535	535	537	1539
vEMINR	4×	**88**	**39**	**96**	**15**	**32**	**27**	**29**	—	—	—	—
	8×	**95**	**50**	**145**	**16**	**41**	**32**	**42**	—	—	—	—
	10×	—	—	—	—	—	—	—	**36**	**34**	**34**	**91**

## 3 Discussion

vEMINR employs a self‐supervised learning approach to transfer knowledge obtained from the high‐resolution (HR) lateral information of anisotropic volumes to the process of recovering missing axial information. Leveraging the continuous representation capability of implicit neural representations, it enables efficient and rapid restoration of volume electron microscopy (vEM) data with arbitrary anisotropy factors. This method improves reconstruction quality by integrating implicit degradation representations of real low‐resolution (LR) images, while also enhancing its robustness in handling domain variations. In our method, we design an implicit degradation extractor that captures global degradation patterns to guide reconstruction and compensate for depth‐related inconsistencies between XY and Z directions, thereby overcoming voxel anisotropy limitations (contrastive learning process of Figure 1a), which distinguishes our approach from the concurrent study [35]. Furthermore our method provides reliable support for downstream tasks such as 3D segmentation and superstructure analysis.

vEMINR can be further optimized and improved. First, we adopted the average pooling method to simulate the axial degradation process of anisotropic volumes. Although this simplified simulation method effectively captures the basic characteristics of axial resolution reduction, it may limit the types of degradation the model can learn. Specifically, average pooling mainly simulates the downsampling process but may not fully represent more complex real‐world degradation scenarios, such as optical blur, electron scattering, or noise introduced during sample preparation. Future work could consider incorporating more complex and diverse degradation models, such as Gaussian blur, Poisson noise, or physics‐based scattering models, to better simulate the various degradation factors in actual imaging processes. Second, in custom experiments, various intermediate steps of vEMINR can be modified, including the feature extraction network structure, weights of implicit degradation representation and loss functions, in order to achieve optimal performance in specific experiments.

In summary, we have validated the ability of vEMINR to perform fast and accurate isotropic reconstruction of vEM data. Experimental results show that vEMINR can effectively reduce the time and hardware requirements in large‐scale vEM data reconstruction. Furthermore, vEMINR demonstrates excellent transferability across different datasets, further proving its broad applicability in practical applications. This not only helps improve research efficiency and reduce costs, but also provides more comprehensive technical support for a deeper understanding of the interactions between organelles within cells.

## 4 Methods

In this section, we first introduce the evaluation metrics used to compare method performance, as well as the relevant datasets. Then, we provide a detailed description of the proposed vEMINR architecture, including the feature extractor, implicit degradation extractor, and implicit neural representation module. Next, we explain the training process of vEMINR and its optimization objectives. Finally, we describe the model's inference procedure.

### 4.1 Metrics and Datasets

#### 4.1.1 Metrics

Our method is compared with the traditional interpolation method, cubic [42] as a baseline. Additionally, we conduct a comprehensive comparison with current mainstream isotropic reconstruction method vEMDiffuse‐a [32]. To objectively evaluate the reconstruction results, we employ multiple quantitative metrics: Structural Similarity Index (SSIM) and Peak Signal‐to‐Noise Ratio (PSNR), which measure the structural similarity and pixel value error between the reconstructed and reference volumes, respectively; Learning Perceptual Patch Similarity (LPIPS) is used to measure the perceptual quality of the reconstructed volume on three orthogonal axis slices. In downstream 3D segmentation tasks, the Dice coefficient is used to evaluate the overlap between the predicted segmentation labels and the ground truth, while the Hausdorff distance is used to assess the boundary accuracy of the segmentation. This comprehensive evaluation verifies the accuracy and reliability of our method in reconstructing the correct 3D structure of biological specimens, and further demonstrates its effectiveness in downstream tasks.

#### 4.1.2 Datasets

We challenge our isotropic reconstruction algorithm on both simulated and real‐world datasets. The simulated datasets are generated by manually downgrading the mouse brain neuron dataset from the EPFL [43] and six public datasets of mouse heart (jrc_mus‐heart), mouse kidney (jrc_mus‐kidney), mouse liver (jrc_mus‐liver, jrc_mus‐liver3), mouse pancreas (jrc_mus‐pancreas), and mouse skin (jrc_mus‐skin) from the OpenOrganelle platform [24] by 4× and 8× along the z‐axis. These diverse and representative datasets allow us to comprehensively evaluate our method in different tissue types. Three publicly available datasets of adult Drosophila brains from Cremi (https://Cremi.org/data/), which were collected using ssTEM with an anisotropy factor of 10, and the Mouse cortical neurons dataset Kasthuri11 [44] are used as real‐world data to further challenge the performance of our method. Detailed information about the datasets used in the experiments can be found in Table S3.

### 4.2 Details of Network Implementation

In this section, we describe the key components of vEMINR in detail: feature extractor, implicit degradation extractor, and implicit neural representation module.

#### 4.2.1 Feature Extractor

Given a low‐resolution image $I_{\text{LR}}\in R^{W\times H\times 1}$ with width W and height H, we employ the Residual Dense Network (RDN) [45] to extract its feature. The RDN comprises the Shallow Feature Extraction Network (SFENet), Residual Dense Blocks (RDBs), and Dense Feature Fusion (DFF).

Shallow Feature Extraction (SFENet). First, the low‐resolution input image $I_{\text{LR}}$ is passed through the Shallow Feature Extraction Network (SFENet) to extract shallow features. SFENet consists of two 3×3 convolutional layers. The first convolutional layer extracts shallow features $F_{-1}$ from $I_{\text{LR}}$:

(1) $$F_{-1}=H_{\text{SFE1}}\left(I_{\text{LR}}\right)$$

where $H_{\text{SFE1}}\left(\cdot \right)$ represents the first convolution operation. These shallow features $F_{-1}$ are then further processed for additional feature extraction and global residual learning. Thus, we obtain:

(2) $$F_{0}=H_{\text{SFE2}}\left(F_{-1}\right)$$

where $H_{\text{SFE2}}\left(\cdot \right)$ denotes the second convolution operation.

Residual Dense Block (RDB). We process $F_{0}$ in Equation (2) through a sequence of d Residual Dense Blocks (RDBs), each consisting of c 3×3 convolutional layers, with the RDBs parameterized by θ. The set of RDBs is parameterized as $\left\{\theta_{1},\theta_{2},\cdots ,\theta_{d}\right\}$, where $\theta_{i}$ represents the parameters of the i‐th RDB. The operation of the i‐th RDB is formulated as:

(3) $$\begin{array}{c} F_{i}=RDB_{\theta_{i}}\left(F_{i-1}\right) \end{array}$$

where i∈{1,2,⋯,d}, $RDB_{\theta_{i}}\left(\cdot \right)$ denotes the i‐th RDB, and $F_{i-1}$ and $F_{i}$ are its input and output, respectively. The final output after processing through all RDBs can be expressed as:

(4) $$\begin{array}{c} F_{d}=RDB_{\theta_{d}}\left(RDB_{\theta_{d-1}}\left(\cdots RDB_{\theta_{1}}\left(F_{0}\right)\right)\right) \end{array}$$

Dense Feature Fusion (DFF). Given the features ($F_{0},F_{1},\cdots ,F_{d}$) extracted by RDBs, we then concatenate these feature maps and perform adaptive global fusion to obtain the global feature map $F_{\text{GFF}}$:

(5) $$F_{\text{GFF}}=H_{\text{GFF}}\left(\left[F_{0},F_{1},\cdots ,F_{d}\right]\right)$$

where $H_{\text{GFF}}\left(\cdot \right)$ is a composite function consisting of a 1×1 convolutional layer (for adaptively fusing features) and a 3×3 convolutional layer (for extracting global features). We then use Global Residual Learning (GRL) to combine the global fusion feature $F_{\text{GFF}}$ in Equation (5) and shallow features $F_{-1}$ in Equation (1) to produce the final output feature map F:

(6) $$F=F_{-1}+F_{\text{GFF}}$$

where $F\in R^{W\times H\times C}$, with width W, height H, and C channels. The total feature extractor process for low‐resolution image $I_{\text{LR}}$ can be formulated as:

(7) $$F=H_{F}\left(I_{\text{LR}}\right)$$

where $H_{F}\left(\cdot \right)$ denotes the feature extractor consisting of SFENet, RDBs, and DFF.

#### 4.2.2 Implicit Degradation Extractor

We also utilize an implicit degradation extractor to process the low‐resolution image $I_{\text{LR}}$ and predict its implicit degradation representation. The implicit degradation extractor consists of two main components: the encoder network and the extractor network.

**Encoder Network**. The encoder network comprises a backbone convolutional neural network (CNN) and a multi‐layer perceptron (MLP) projection head. The backbone CNN serves as a feature extractor and consists of six consecutive convolutional layers. Given the low‐resolution image $I_{\text{LR}}$, the feature extraction process through the backbone CNN can be represented as:

(8) $$F_{e}=H_{c}\left(I_{\text{LR}}\right)$$

where $H_{c}\left(\cdot \right)$ represents the operations of the backbone CNN, and $F_{e}$ denotes the extracted feature. We then compress the feature map $F_{e}$ by adaptive average pooling and flattening operations:

(9) $$V=\text{Flatten}(H_{\text{AvgPool}}\left(F_{e}\right))$$

where $H_{\text{AvgPool}}\left(\cdot \right)$ and Flatten(·) represent the adaptive average pooling and flattening operations, respectively, and $V\in R^{C}$ is the output vector in C dimensions. We next adopt the projection head consisting of three fully connected layers to further process the vector V in Equation (9):

(10) $$Z=H_{\text{pro}}\left(V\right)$$

where $H_{\text{pro}}\left(\cdot \right)$ represents the projection operation, $Z\in R^{C}$ denotes the image representation. The entire encoding process from the low‐resolution image $I_{\text{LR}}$ to the above representation, Z can be formulated as:

(11) $$Z=H_{Z}\left(I_{\text{LR}}\right)$$

where $H_{Z}\left(\cdot \right)$ represents the encoder operations, including the backbone CNN and MLP projection head.

**Extractor Network**. To avoid letting the model collapse into a trivial solutions, we introduce an extractor network to process the representation Z in Equation (11). The extractor network consists of two fully connected layers and its process can be represented as:

(12) $$P=H_{\text{pre}}\left(Z\right)$$

where $H_{\text{pre}}\left(\cdot \right)$ is the projection operation in the extractor, and $P\in R^{C}$ is the implicit degradation representation. Based on Equations (11) and (12), the process in the implicit degradation extractor from the low‐resolution image $I_{\text{LR}}$ to P is given by:

(13) $$P=H_{\text{pre}}\left(H_{Z}\left(I_{\text{LR}}\right)\right)$$

where $H_{Z}\left(\cdot \right)$ and $H_{\text{pre}}\left(\cdot \right)$ denote the operations of the encoder and extractor networks, respectively.

#### 4.2.3 Implicit Neural Representation

In this section, we employ the implicit neural representation method [34] to reconstruct the super‐resolution image $I_{\text{SR}}\in R^{W\times H\times 1}$. This reconstruction process integrates the implicit degradation representation ($P\in R^{C}$, as defined in Equation (13)), the feature map of the low‐resolution image ($F\in R^{W\times H\times C}$, as defined in Equation (7)), and the 2D coordinate map corresponding to the target dimensions, width W and height H. The inclusion of the 2D coordinate map is critical, as it facilitates the reconstruction of $I_{\text{SR}}$ by enabling pixel‐wise prediction of intensity values based on the spatial positions defined in the map.

In the following, we describe the pixel‐wise reconstruction process for super‐resolution image $I_{\text{SR}}$. To determine the intensity of $I_{\text{SR}}$ at the coordinate (i,j), where 0≤i≤W−1 and 0≤j≤H−1, we obtain its encoded coordinate ($\gamma_{i,j}$), extract its region information (C), and retrieve the corresponding feature (n) in the low‐resolution image's feature map (F in Equation (7)). These components, along with the implicit degradation representation (P in Equation (13)), are concatenated and input into the implicit neural representation module to predict the intensity value:

(14) $$I_{\text{SR}}\left(i,j\right)=f_{\vartheta }\left(\gamma_{i,j},C,n,P\right)$$

where $f_{\vartheta }\left(\cdot \right)$ is the implicit neural representation module parameterized by a multi‐layer perceptron, and $I_{\text{SR}}\left(i,j\right)$ is the intensity value of the super‐resolution image $I_{\text{SR}}$ at coordinate (i,j).

Specifically, among the inputs to the implicit neural representation module, the encoded coordinate $\gamma_{i,j}$ is obtained by applying the Gaussian Fourier feature mapping to the coordinate vector [i,j]:

(15) $$\gamma_{i,j}=\left[\begin{array}{c} \cos(2\pi B[i,j])\\ \sin(2\pi B[i,j]) \end{array}\right]$$

where the matrix $B\in R^{m\times 2}$, with m representing the feature map dimensionality, contains entries independently sampled from a Gaussian distribution $N(0,\sigma^{2})$, and cos(·) and sin(·) denote element‐wise cosine and sine operations, respectively. The region information (C) can be formulated as:

(16) $$C=[c_{w},c_{h}]$$

where $c_{w}$ and $c_{h}$ represent the width and height of the regions defined by the target pixel positions in the coordinate map. For the coordinate (i,j), its corresponding feature (n) is obtained by querying the feature map of the low‐resolution image (F in Equation (7)). We first assign feature vectors in this map in the 2D continuous domain of $I_{\text{SR}}$, and then obtain the nearest feature vector for coordinate (i,j):

(17) $$\begin{array}{c} n=Q(F,i,j) \end{array}$$

where Q(·) denotes the feature querying process for coordinate (i,j). The other input to the implicit neural representation module, namely P, is the implicit degradation representation as defined in Equation (12).

During the super‐resolution image reconstruction process ($I_{\text{SR}}$), we utilize the implicit neural representation module to predict the intensity values of all pixel coordinates through W×H iterations:

(18) $$I_{\text{SR}}=\left\{I_{\text{SR}}\left(i,j\right)|i\in \left[0,W-1\right],j\in \left[0,H-1\right]\right\}$$

To formalize the above reconstruction process, we express it as:

(19) $$I_{\text{SR}}=F\left(F,P\right)$$

where F(·) denotes the reconstruction process using the implicit neural representation module.

### 4.3 Training Process

In this section, we describe in detail the training data preparation method and the optimization objectives of each module of vEMINR.

#### 4.3.1 Training Data Preparation

During the training of vEMINR, we apply average pooling to manually degrade the high‐resolution (HR) transverse (XY) slices of the anisotropic volume to generate simulated low‐resolution (LR) images as input. Specifically, given an HR transverse slice $I_{\text{HR}}\in R^{W\times H\times 1}$ from an anisotropic volume $V_{\text{aniso}}\in R^{W\times H\times L_{\text{aniso}}\times 1}$ with dimensions W, H, and $L_{\text{aniso}}$, we degrade $I_{\text{HR}}$ into an LR slice $I_{\text{LR}}$ by a given anisotropy factor x. The degradation process is defined as follows:

(20) $$I_{\text{LR}}=\left\{\begin{array}{cc} \frac{1}{x}\sum_{m=0}^{x-1}I_{\text{HR}}\left(w+m,h\right), & \text{for degradation along the X-axis},\\ \frac{1}{x}\sum_{m=0}^{x-1}I_{\text{HR}}\left(w,h+m\right), & \text{for degradation along the Y-axis} \end{array}\right.$$

where w and h denote the indices of $I_{\text{HR}}$ along the X‐axis and Y‐axis, respectively. For degradation along the X‐axis, $I_{\text{LR}}\in R^{W\times H\times 1}$ satisfies $W=\frac{W}{x}$ and H=H; for degradation along the Y‐axis, $I_{\text{LR}}\in R^{W\times H\times 1}$ satisfies W=W and $H=\frac{H}{x}$.

#### 4.3.2 Optimization Objective of Implicit Degradation Extractor

We use the SiamSim [46] self‐supervised contrastive learning to train the implicit degradation extractor. Given two augmented views ($I_{\text{LR}}^{1}$ and $I_{\text{LR}}^{2}$) cropped from the low‐resolution image $I_{\text{LR}}$ in Equation (20), the implicit degradation extractor extracts their features $Z_{1}$, $Z_{2}$, and implicit degradation representations $P_{1}$, $P_{2}$ as described in Equations (11) and (12):

(21) $$\left\{\begin{array}{cc} Z_{1}=H_{Z}\left(I_{\text{LR}}^{1}\right); & P_{1}=H_{\text{pre}}\left(Z_{1}\right),\\ Z_{2}=H_{Z}\left(I_{\text{LR}}^{2}\right); & P_{2}=H_{\text{pre}}\left(Z_{2}\right) \end{array}\right.$$

The contrastive learning optimization objective is to minimize the negative cosine similarity between these encoded features and the implicit degenerate representation:

(22) $$\left\{\begin{array}{c} D\left(P_{1},Z_{2}\right)=-\frac{P_{1}}{∥P_{1}{∥}_{2}}\cdot \frac{Z_{2}}{∥Z_{2}{∥}_{2}},\\ D\left(P_{2},Z_{1}\right)=-\frac{P_{2}}{∥P_{2}{∥}_{2}}\cdot \frac{Z_{1}}{∥Z_{1}{∥}_{2}} \end{array}\right.$$

where ${∥\cdot ∥}_{2}$ represents $\ell_{2}$ normalization.

Based on the above negative cosine similarity, the contrastive learning loss function for our implicit degradation extractor can be formulated as:

(23) $$L_{\text{contrast}}=\frac{1}{2}D\left(P_{1},Z_{2}\right)+\frac{1}{2}D\left(P_{2},Z_{1}\right)$$

To prevent network collapse, we apply the ‘stop‐gradient’ operation to the feature vectors ($Z_{1}$, $Z_{2}$), treating them as constants in the loss function and preventing gradient updates, thus ensuring training stability. By incorporating the ‘stop‐gradient’ operation, the loss function in Equation (23) is reformulated as:

(24) $$L_{\text{contrast}}=\frac{1}{2}D\left(P_{1},\text{stopgrad}\left(Z_{2}\right)\right)+\frac{1}{2}D\left(P_{2},\text{stopgrad}\left(Z_{1}\right)\right)$$

After the contrastive learning process, the implicit degradation extractor can effectively learn visual representations of low‐resolution images.

#### 4.3.3 Optimization Objective of Feature Extractor and INR

The joint training loss for the feature extractor and INR is defined as the L1 loss between the SR image $I_{\text{SR}}$ (Equation (19)) and the HR image $I_{\text{HR}}$:

(25) $$L_{\text{SR}}=\frac{1}{W\times H}\sum_{W,H}\left|I_{\text{SR}}-I_{\text{HR}}\right|$$

where W and H represent the width and height of the SR image $I_{\text{SR}}$, and the HR image $I_{\text{HR}}$, respectively.

### 4.4 Inference Process

In this section, we outline the inference process of vEMINR, which utilizes the vEMINR method to reconstruct the isotropic volume from the anisotropic volume. The anisotropic volume $V_{\text{aniso}}\in R^{W\times H\times L_{\text{aniso}}\times 1}$ can be regarded as a collection of low‐resolution slices obtained by division along either the X‐ or Y‐axis, as shown below:

(26) $$V_{\text{aniso}}=\left\{\begin{array}{cc} \{I_{\text{LR-YZ}}^{0},I_{\text{LR-YZ}}^{1},\text{…},I_{\text{LR-YZ}}^{i},\text{…},I_{\text{LR-YZ}}^{W-1}\}, & \text{or}\\ \left\{I_{\text{LR-XZ}}^{0},I_{\text{LR-XZ}}^{1},\text{…},I_{\text{LR-XZ}}^{j},\text{…},I_{\text{LR-XZ}}^{H-1}\right\} & \end{array}\right.$$

where $I_{\text{LR-YZ}}$ and $I_{\text{LR-XZ}}$ represent the low‐resolution slices along the X‐ and Y‐axes, respectively, and the superscript i and j denote the slice indexes. Based on the aforementioned low‐resolution slices, we employ a feature extractor to obtain the feature map (as shown in Equation (7)) and use the implicit degradation extractor to derive the implicit degradation representation (as outlined in Equation (13)):

(27) $$\left\{\begin{array}{cc} F_{YZ}^{i}=H_{F}\left(I_{\text{LR-YZ}}^{i}\right), & P_{YZ}^{i}=H_{\text{pre}}\left(H_{Z}\left(I_{\text{LR-YZ}}^{i}\right)\right),\;0\leq i\leq W-1,\\ F_{XZ}^{j}=H_{F}\left(I_{\text{LR-XZ}}^{j}\right), & P_{XZ}^{j}=H_{\text{pre}}\left(H_{Z}\left(I_{\text{LR-XZ}}^{j}\right)\right),\;0\leq j\leq H-1 \end{array}\right.$$

where $F_{YZ}^{i}$ and $F_{XZ}^{j}$ denote the feature maps of $I_{\text{LR-YZ}}^{i}$ and $I_{\text{LR-XZ}}^{j}$, respectively, and $P_{YZ}^{i}$ and $P_{XZ}^{j}$ represent their corresponding implicit degradation representations. We then employ the implicit neural representation module to reconstruct the super‐resolution images (see Equation (19)):

(28) $$\begin{array}{cc} & I_{\text{SR-YZ}}^{i}=F\left(F_{YZ}^{i},P_{YZ}^{i}\right),\;I_{\text{SR-XZ}}^{j}=F\left(F_{XZ}^{j},P_{XZ}^{j}\right),\\ & 0\leq i\leq W-1,\;0\leq j\leq H-1 \end{array}$$

where $I_{\text{SR-YZ}}^{i}$ and $I_{\text{SR-XZ}}^{j}$ represent the reconstructed super‐resolution images corresponding to $I_{\text{LR-YZ}}^{i}$ and $I_{\text{LR-XZ}}^{j}$, respectively. We next perform 2D stitching the operation and 3D registration and reconstruct the super‐resolution images into the 3D volumes ($V_{\text{YZ}}$ and $V_{\text{XZ}}$):

(29) $$\left\{\begin{array}{c} V_{\text{YZ}}=\left\{I_{\text{SR-YZ}}^{0},I_{\text{SR-YZ}}^{1},\text{…},I_{\text{SR-YZ}}^{i},\text{…},I_{\text{SR-YZ}}^{W-1}\right\}\\ V_{\text{XZ}}=\left\{I_{\text{SR-XZ}}^{0},I_{\text{SR-XZ}}^{1},\text{…},I_{\text{SR-XZ}}^{j},\text{…},I_{\text{SR-XZ}}^{H-1}\right\} \end{array}\right.$$

Finally, we reconstruct the isotropic volume by averaging the above volumes:

(30) $$V_{\text{iso}}=avg\left(V_{\text{XZ}},V_{\text{YZ}}\right)$$

where $V_{\text{iso}}\in R^{W\times H\times L_{\text{iso}}\times 1}$ denotes the isotropic 3D volume, and avg(·) denotes the weighted average operation between the corresponding voxels of two 3D volumes.

## Author Contributions

Renmin Han conceived the idea and supervised the experimental design and manuscript revisions. Wenjia Meng contributed to the algorithm design, supported the implementation, and assisted in the writing of the manuscript. Jibin Yang contributed to code development, experimental design and implementation, as well as manuscript writing and revisions. Muyu Liu contributed to code development. Jie Huo provided guidance on manuscript revisions and experimental adjustments. Yan Zhang offered assistance and advice on experimental design. Chenjie Feng and Gang Pan contributed to manuscript revisions.

## Conflicts of Interest

The authors declare no conflicts of interest.

## Code Availability

The source codes, training, and inference notebooks of vEMINR are available at https://github.com/KysonYang001/vEMINR.git.

## Supporting information




**Supporting File**: advs73520‐sup‐0001‐SuppMat.pdf.

## Data Availability

The data that support the findings of this study are available in the Supporting Information of this article.
