# Supplementary material for: vEMINR: Ultra‐Fast Isotropic Reconstruction for Volume Electron Microscopy With Implicit Neural Representation
Source: Adv Sci (Weinh). 2026 Jan 30;13(13):e11922. doi: 10.1002/advs.202511922 (PMC12955999; doi:10.1002/advs.202511922)
Supplement: Supplementary file 1 — Supporting File: advs73520‐sup‐0001‐SuppMat.pdf. [file ADVS-13-e11922-s001.pdf]

vEMINR: Ultra-Fast Isotropic Reconstruction for Volume Electron Microscopy with Implicit Neural Representation

Jibin Yang[1,2,3†], Jie Huo[4,†], Muyu Liu[1,†], Chenjie Feng[3], Yan Zhang[5], Gang Pan[6], Wenjia Meng[1,*], Renmin Han[2,3,7*]

[1]School of Software, Shandong University, 1500 Shunhua Road, Jinan 250101, China; [2]Frontiers Science Center for Nonlinear Expectations (Ministry of Education); Research Center for Mathematics and Interdisciplinary Sciences, Shandong University, Qingdao 266237, China; [3]College of Medical Information and Engineering, Ningxia Medical University, Yinchuan 750004, China; [4]School of Physics, Ningxia University, 217 Wencui North Street, Yinchuan 750021, China; [5]National Laboratory of Biomacromolecules; Institute of Biophysics, Chinese Academy of Sciences, Bei jing 100101, China; [6]The State Key Lab of Brain-Machine Intelligence, Zhejiang University, Hangzhou 310027, China; [7]Shanghai YueXin Lifescience Information Technology Company, 100 Qinzhou Road, Shanghai 200235, China

1 Supplementary demonstrations of vEMINR performance

To demonstrate the ability of our isotropic reconstruction method to handle complex and diverse cellular scenes, we conducted 4× and 8× reconstruction experiments on seven simulated datasets and evaluated the results. These datasets cover a variety of tissues, including mouse brain, mouse liver, mouse pancreas, mouse skin, mouse heart, and mouse kidney. Since there is no significant visual difference in the 4× reconstruction results, we only present the 8× reconstruction results (Figure S1 and Figure S2). As shown by the red arrows in figures, vEMINR achieves the best structural detail recovery compared to the other two methods in the isotropic reconstruction task of simulated data from different sources. This result confirms that our method can effectively match axial degradation types and perform

[†]These authors should be regarded as Joint First Authors; [*]All correspondence should be addressed to Wenjia Meng (wjmeng@sdu.edu.cn) and Renmin Han (hanrenmin@sdu.edu.cn).

reliable reconstructions.

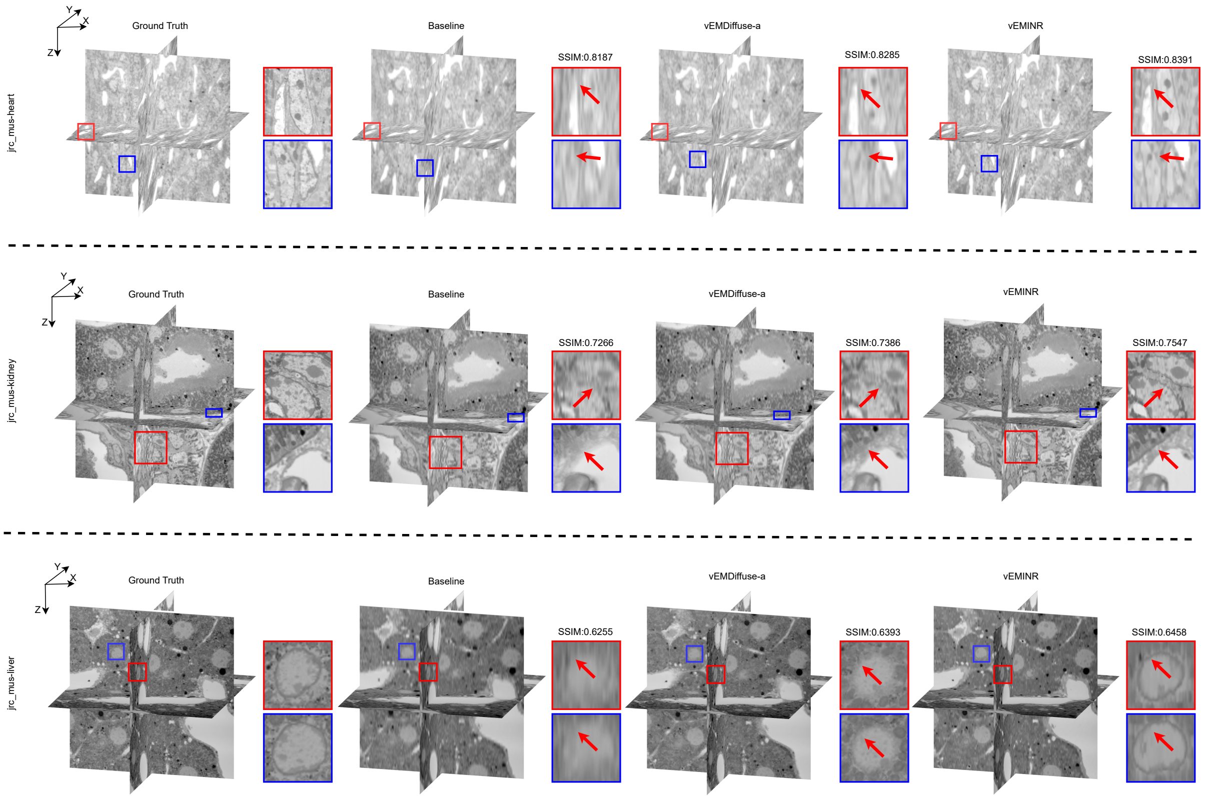

Figure S1: 3D visualization of anisotropic reconstruction results from different methods on jrc_mus-heart, jrc_mus-kidney and jrc_mus-liver datasets (anisotropy factor of 8).

We quantified the difference between the reconstructed volumes and the ground truth volumes using SSIM and PSNR (Table S1). It can be seen that vEMINR achieved the best evaluation results in all quantitative assessments. In the 4× reconstruction experiment, the PSNR increased by an average of approximately 2.1% to 6.5%, while the SSIM improved by an average of approximately 2.3% to 6.4%. In the higher 8× reconstruction experiment, the PSNR increased by an average of approximately 2.2% to 3.1%, and the SSIM improved by an average of approximately 1.4% to 4.3%. We further calculated the SSIM, PSNR, and LPIPS metrics for the three orthogonal slices (XY, YZ, and XZ) of the reconstructed volume compared to the ground truth (Figure S3, Figure S4, Figure S5, Figure S6). The results show that vEMINR consistently maintains the best reconstruction performance.

To demonstrate the positive impact of our method on downstream 3D segmentation tasks, we performed 3D segmentation on the volumes 8× reconstructed from different simulated datasets (Figure S7). Compared to other methods,

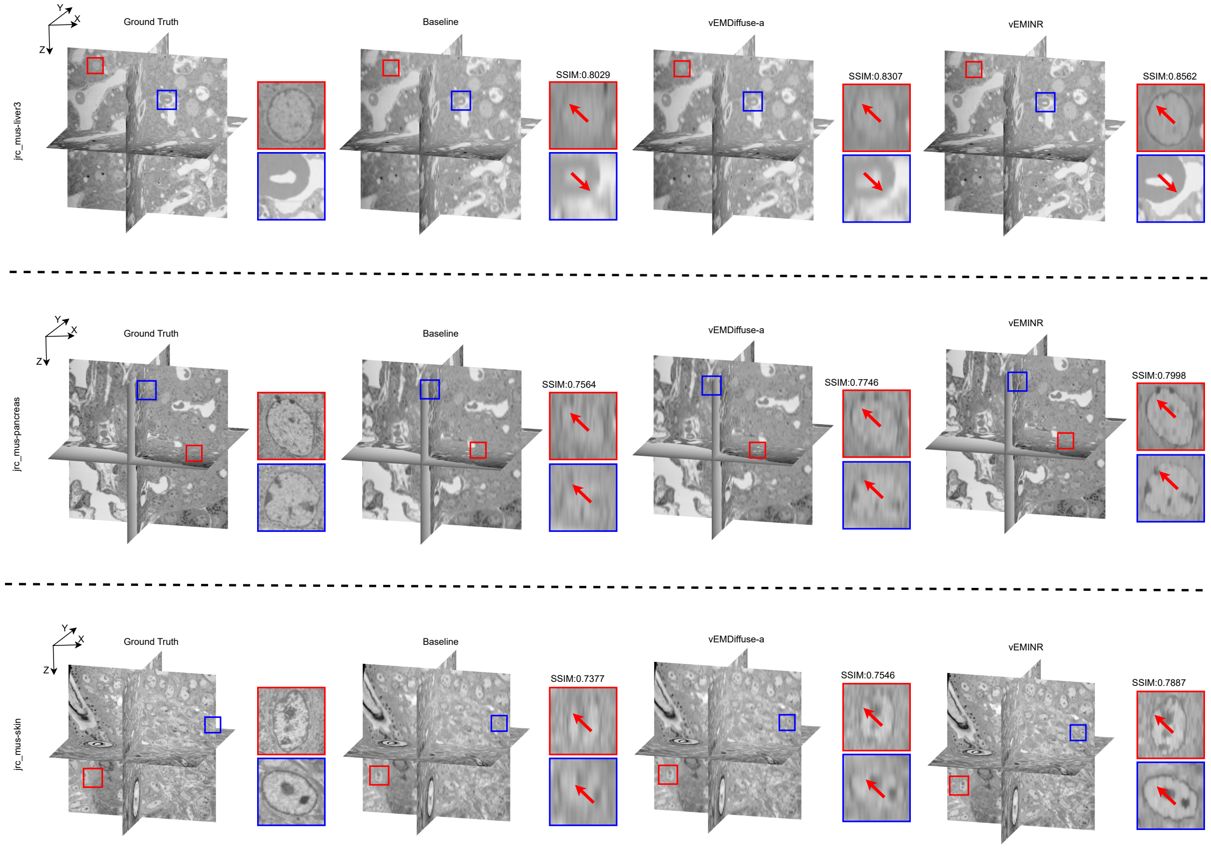

Figure S2: 3D visualization of anisotropic reconstruction results from different methods on jrc_mus-liver3, jrc_mus-pancreas and jrc_mus-skin datasets (anisotropy factor of 8).

Table S1: Results of quantitative comparison of isotropic reconstruction volumes of different methods

Datasets	Metric	Scale Factor 4x			Scale Factor 8x		
		Baseline	vEMDiffuse-a	vEMINR	Baseline	vEMDiffuse-a	vEMINR
jrc_mus-heart	PSNR	35.79	36.32	**36.75**	30.66	30.96	**31.61**
	SSIM	0.9276	0.9396	**0.9441**	0.8187	0.8285	**0.8391**
jrc_mus-kidney	PSNR	29.79	31.59	**32.68**	27.35	27.69	**28.38**
	SSIM	0.8072	0.8676	**0.8910**	0.7266	0.7386	**0.7547**
jrc_mus-liver	PSNR	31.65	32.07	**32.32**	26.55	26.65	**27.03**
	SSIM	0.8288	0.8391	**0.8563**	0.6255	0.6393	**0.6458**
jrc_mus-liver3	PSNR	33.03	35.79	**36.95**	31.52	32.04	**33.28**
	SSIM	0.8496	0.8729	**0.9298**	0.8029	0.8437	**0.8562**
jrc_mus-pancreas	PSNR	32.39	33.52	**33.91**	28.94	29.20	**29.64**
	SSIM	0.8630	0.8875	**0.8978**	0.7564	0.7896	**0.7998**
jrc_mus-skin	PSNR	29.35	31.87	**32.86**	31.55	31.71	**32.41**
	SSIM	0.7876	0.8581	**0.8908**	0.7337	0.7759	**0.7887**

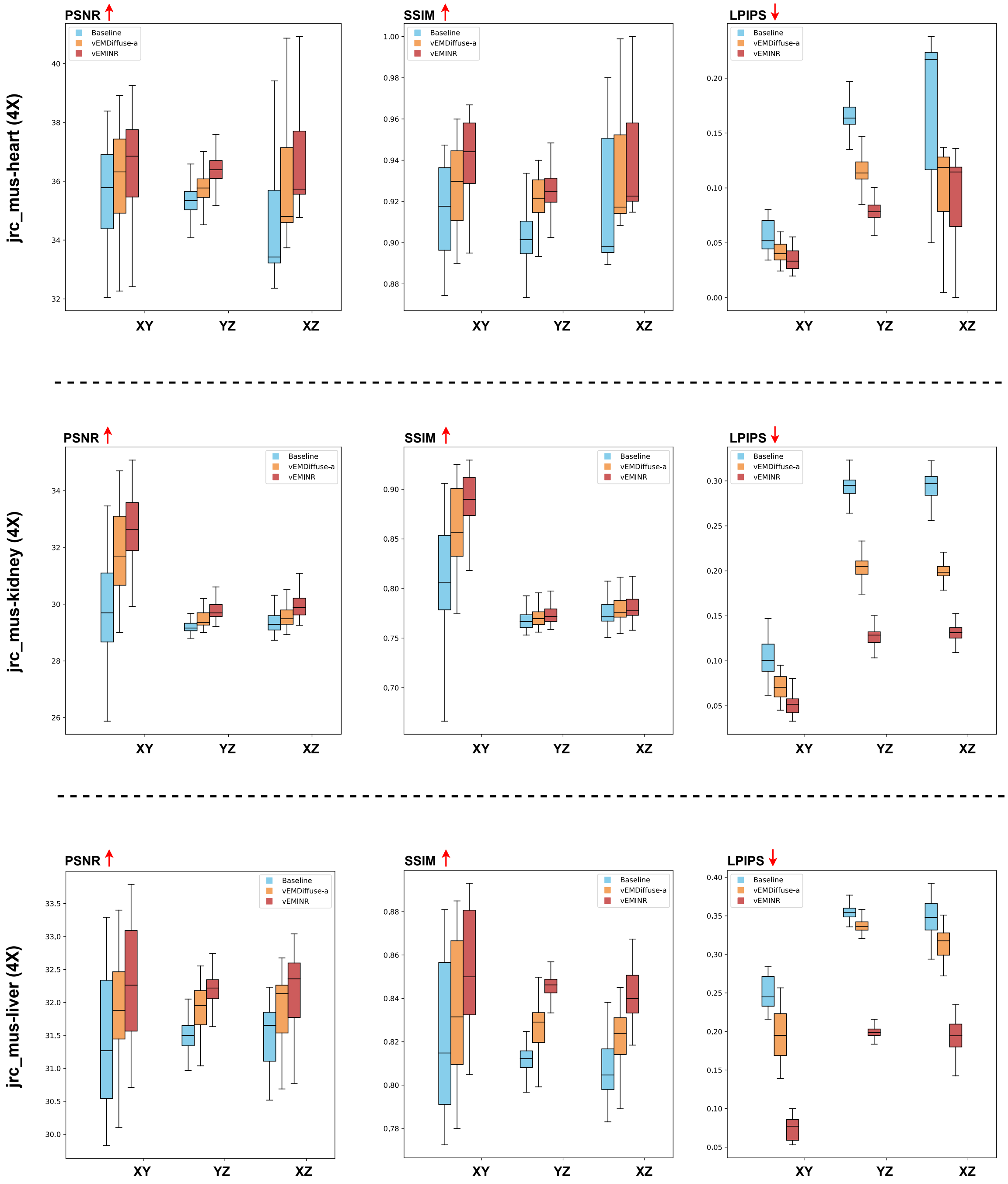

Figure S3: Box plots of PSNR, SSIM, and LPIPS metrics for reconstruction results of jrc_mus-heart,jrc_mus-kidney, and jrc_mus-liver across various methods in XY, YZ, and XZ planes (anisotropy factor of 4).

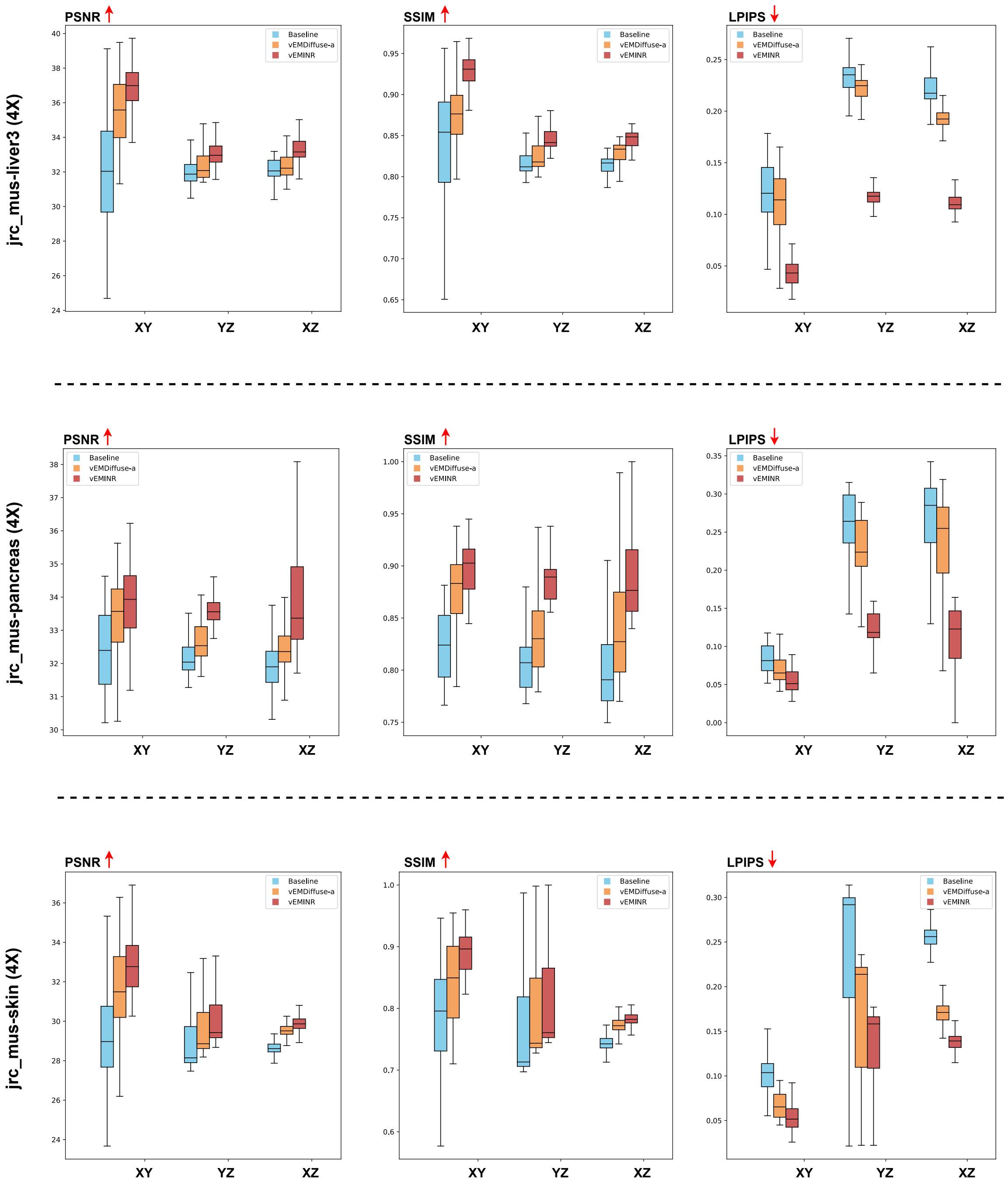

Figure S4: Box plots of PSNR, SSIM, and LPIPS metrics for reconstruction results of jrc_mus-liver3, jrc_mus-pancreas, and jrc_mus-skin across various methods in XY, YZ, and XZ planes (anisotropy factor of 4).

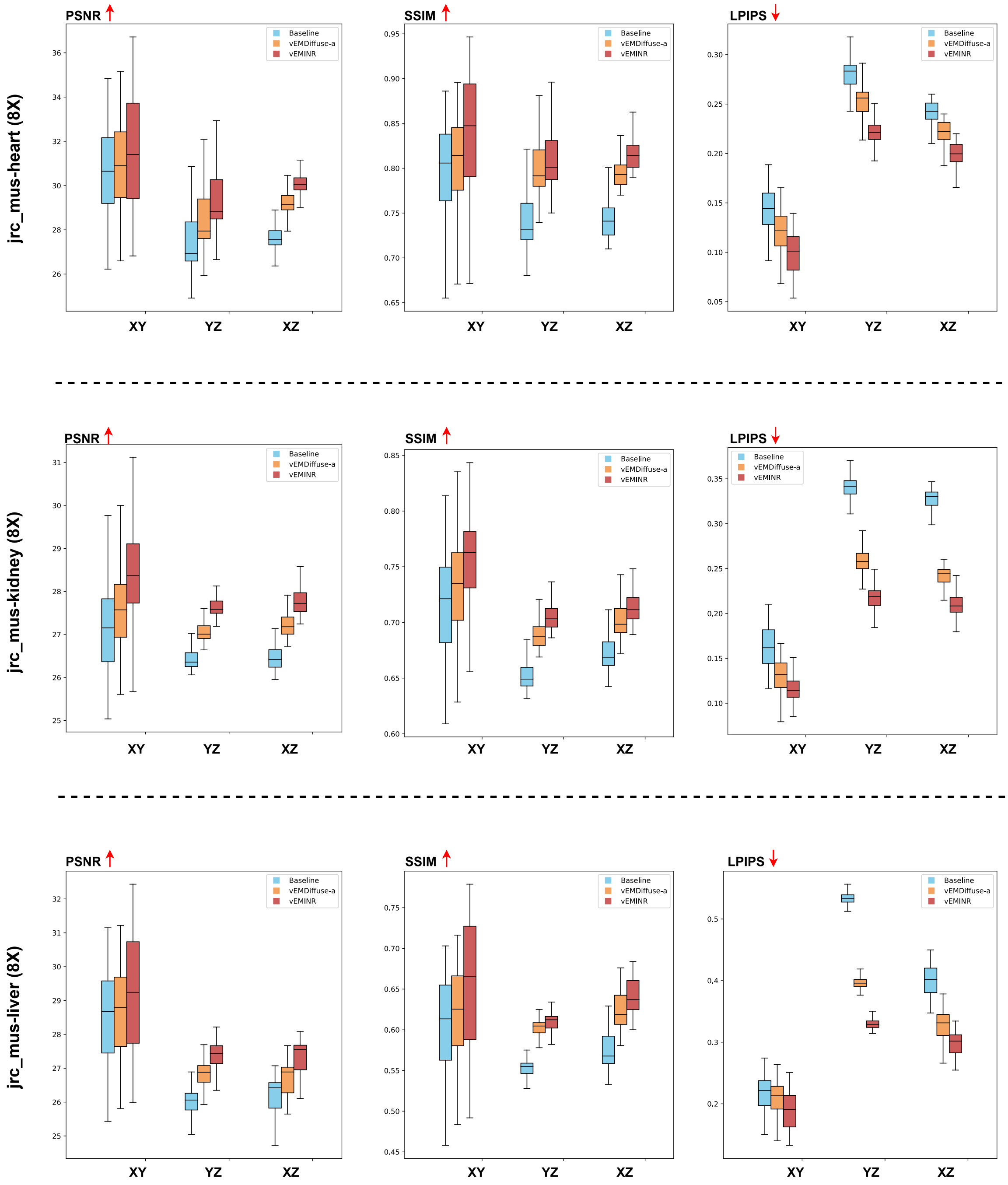

Figure S5: Box plots of PSNR, SSIM, and LPIPS metrics for reconstruction results of jrc_mus-heart,jrc_mus-kidney, and jrc_mus-liver across various methods in XY, YZ, and XZ planes (anisotropy factor of 8).

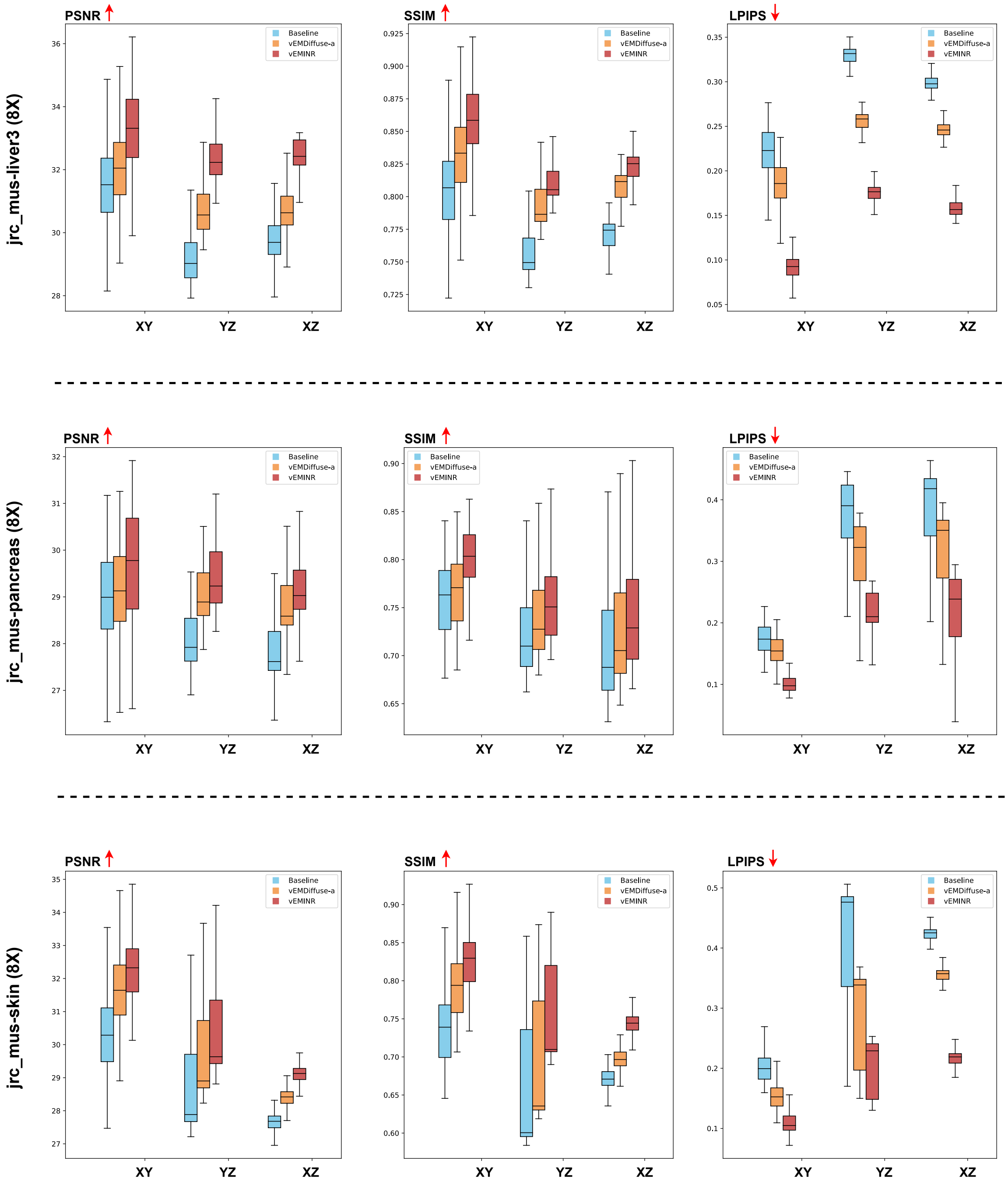

Figure S6: Box plots of PSNR, SSIM, and LPIPS metrics for reconstruction results of jrc_mus-liver3, jrc_mus-pancreas, and jrc_mus-skin across various methods in XY, YZ, and XZ planes (anisotropy factor of 8).

our approach generates the most visually accurate results, effectively preserving the correct 3D structure of biological samples. Furthermore, we quantified the segmentation results using Dice Coefficient and Hausdorff distance (Table S2). The results show that vEMINR achieved the highest Dice coefficient and the lowest Hausdorff distance across all simulated datasets. These results further confirm that vEMINR can more accurately reconstruct the 3D structures of biological samples, which is crucial for downstream tasks that utilize these reconstructed volumes.

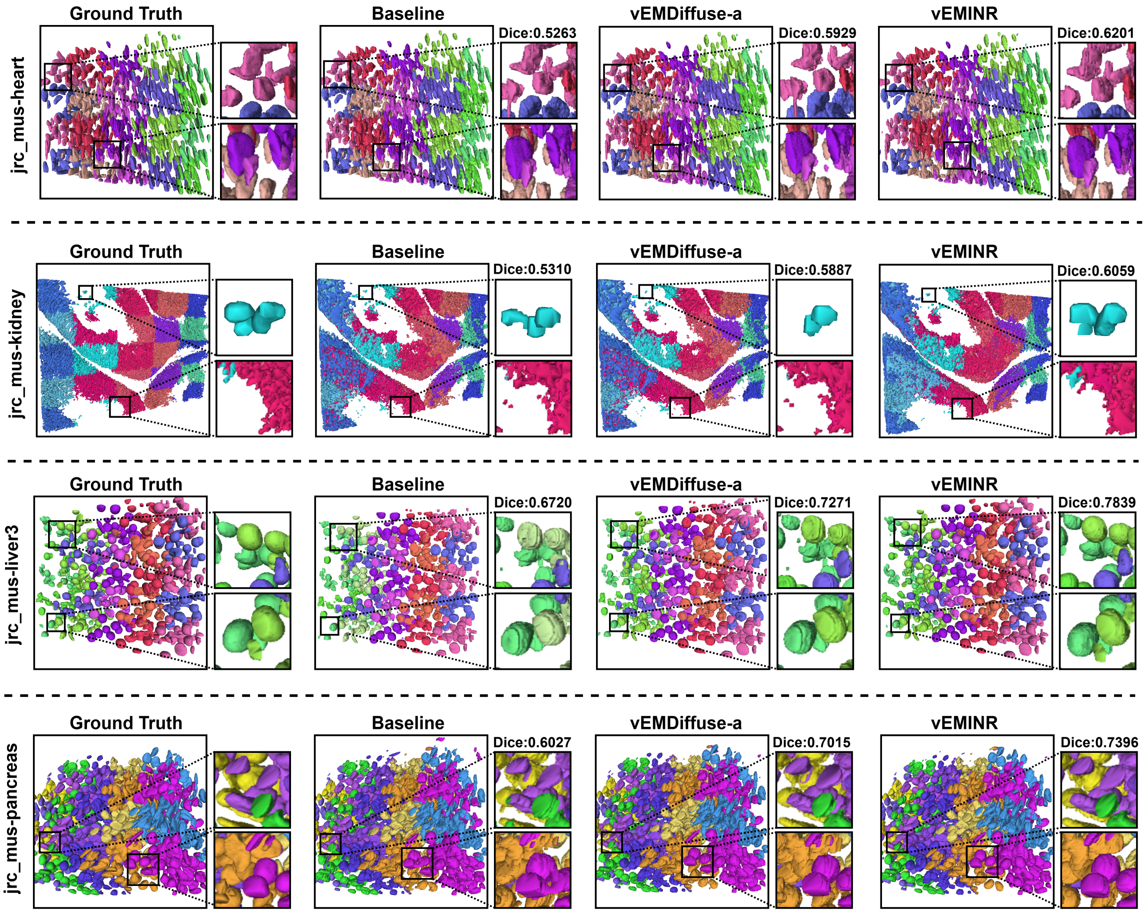

Figure S7: 3D segmentation visualization of isotropic reconstruction results for various methods on jrc_mus-heart, jrc_mus-kidney, jrc_mus-liver3 and jrc_mus-pancreas datasets.

Table S2: 3D segmentation metrics on 8× reconstructed volumes for various methods.

Dataset	Metric	Baseline	vEMDiffuse-a	vEMINR
jrc_mus-heart	Dice Coefficient ↑	0.5035	0.5929	**0.6201**
	Hausdorff Distance ↓	151.32	94.46	**72.06**
jrc_mus-kidney	Dice Coefficient ↑	0.5310	0.5887	**0.6059**
	Hausdorff Distance ↓	174.68	124.68	**106.77**
jrc_mus-liver	Dice Coefficient ↑	0.6411	0.7594	**0.7863**
	Hausdorff Distance ↓	205.71	135.25	**92.56**
jrc_mus-liver3	Dice Coefficient ↑	0.6720	0.7271	**0.7839**
	Hausdorff Distance ↓	177.28	115.59	**78.42**
jrc_mus-pancreas	Dice Coefficient ↑	0.6027	0.7015	**0.7396**
	Hausdorff Distance ↓	212.43	104.32	**76.77**
jrc_mus-skin	Dice Coefficient ↑	0.5938	0.7199	**0.7638**
	Hausdorff Distance ↓	162.03	102.30	**72.37**

2 Details of datasets

We selected six publicly available datasets from the OpenOrganelle platform [1] and the FIB-SEM dataset from EPFL for simulation [2]. These datasets include various vEM volumetric data from different mouse tissues, including heart, kidney, liver, skin, pancreas, and brain. With these high-resolution images as ground truth, we can comprehensively assess the applicability and robustness of our methods across different biological tissues. In terms of real-world data, we obtained three adult Drosophila melanogaster brain datasets collected by the ssTEM method from the CREMI repository. These datasets contains 125 TEM images with a resolution of $4 \times 4 \times 40$ nm/voxel. By conducting extensive experiments on real anisotropic volumes, we validated the performance and generalization capability of vEMINR in reconstructing real anisotropic data. Table S3 provides detailed information about the datasets used in the

experiments.

Table S3: Details of the Data Used in the Experiment

Datasets	Image Shape	Number of Slices	URL
jrc_mus-heart-1-s4	1275×1286	1061	https://open.quiltdata.com/b/janelia-cosem-datasets/tree/jrc_mus-heart-1
jrc_mus-kidney-s4	767×498	1387	https://open.quiltdata.com/b/janelia-cosem-datasets/tree/jrc_mus-kidney
jrc_mus-liver-3-s4	1135×898	1127	https://open.quiltdata.com/b/janelia-cosem-datasets/tree/jrc_mus-liver-3
jrc_mus-liver-s4	796×795	558	https://open.quiltdata.com/b/janelia-cosem-datasets/tree/jrc_mus-liver
jrc_mus-pancreas-4-s4	1142×883	898	https://open.quiltdata.com/b/janelia-cosem-datasets/tree/jrc_mus-pancreas-4
jrc_mus-skin-1	1081×892	1231	https://open.quiltdata.com/b/janelia-cosem-datasets/tree/jrc_mus-skin-1
EPFL	2048×1536	1065	https://www.epfl.ch/labs/cvlab/data/data-em
CREMI Dataset A	1250×1250	125	https://cremi.org/data
CREMI Dataset B	1250×1250	125	https://cremi.org/data
CREMI Dataset C	1250×1250	125	https://cremi.org/data
Kasthuri11	11776×13824	928	https://neurodata.io/data/kasthuri15/

3 Network structure

We adopt the Residual Dense Network (RDN) [3] as a feature extractor to extract features from low-resolution (LR) images (Figure S8). This network consists of two 3×3 convolutional layers for shallow feature extraction, followed by 16 Residual Dense Blocks (RDBs) for further feature extraction (each RDB contains 8 layers of 3×3 convolutional layers for feature extraction and one 1×1 convolutional layer for local feature fusion) and ends with a 1×1 convolutional layer and a 3×3 convolutional layer for global feature fusion. In this process, local and global residual learning are implemented through dense connections.

Our implicit degradation predictor consists of an Encoder and a Predictor (Figure S9). The Encoder includes 6 layers of 3×3 convolutional layers for feature extraction and a 3-layer MLP as the head network. The Predictor is a 2-layer MLP that takes the output of the Encoder as input to generate the final predicted implicit degradation representation. This module is trained using the unsupervised contrastive learning method [4].

The outputs of the feature extractor and the implicit degradation predictor are fed together into the implicit neural representation for upsampling (Figure S10). This module parameterizes the decoding function as a 6-layer MLP, which performs interpolation on the corresponding pixel positions for any given spatial coordinates [5].

In the isotropic reconstruction experiments, we evaluate the usability of the reconstruction results in downstream tasks by segmenting them in 3D. We use the excellent performance image segmentation model [6] to segment the reconstruction results, and the structure of the segmentation network is shown in Figure S11.

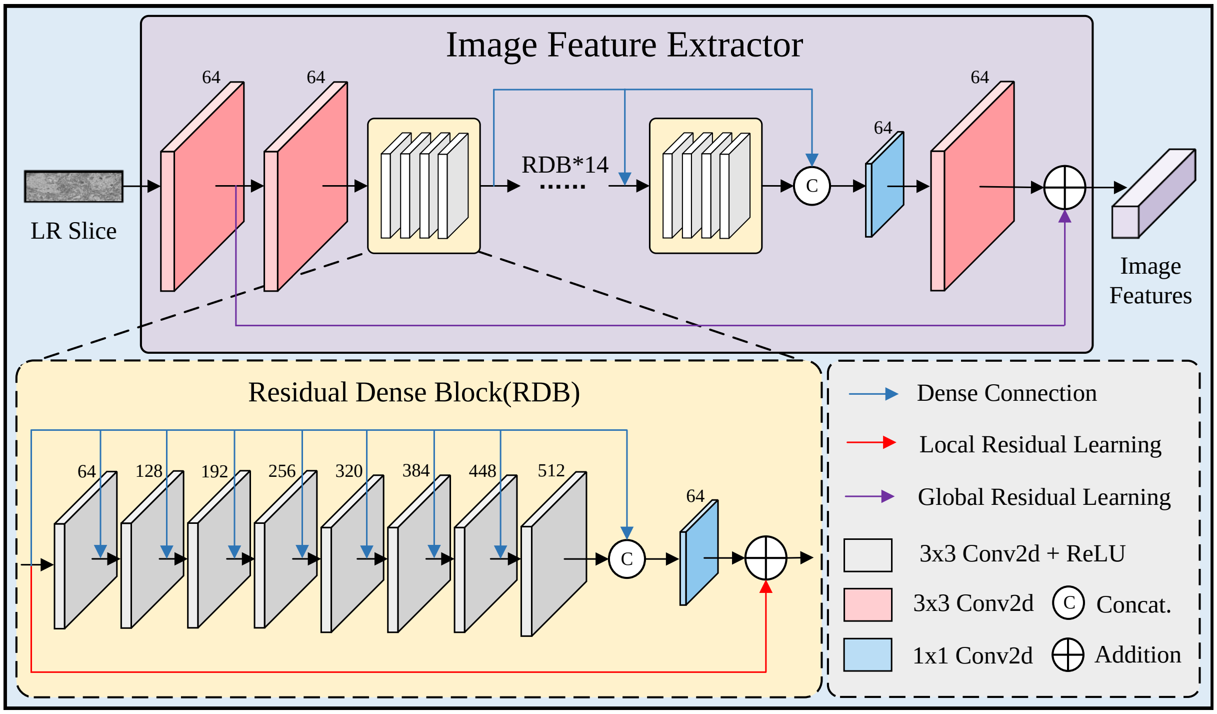

Figure S8: The network structure of image feature extractor for extracting low-resolution image features.

4 Training details

For vEMINR, we train it using all the datasets mentioned in Section 2. To enhance the training set, we cropped all the training slices into many smaller patches of size 48×48. For the comparison method vEMDiffuse-a [7], we use the same datasets and followed the training procedures described in their respective papers.

Regarding the segmentation network shown in Figure S11, we trained a separate segmentation model for each test dataset from OpenOrganelle using the ground truth and corresponding segmentation labels. Specifically, for training each data type, we set the batch size to 4, the learning rate to 1×10^{-4}, and used the Adam optimizer. All other hyperparameters were kept at their default settings, and the model was trained for a total of 500 epochs.

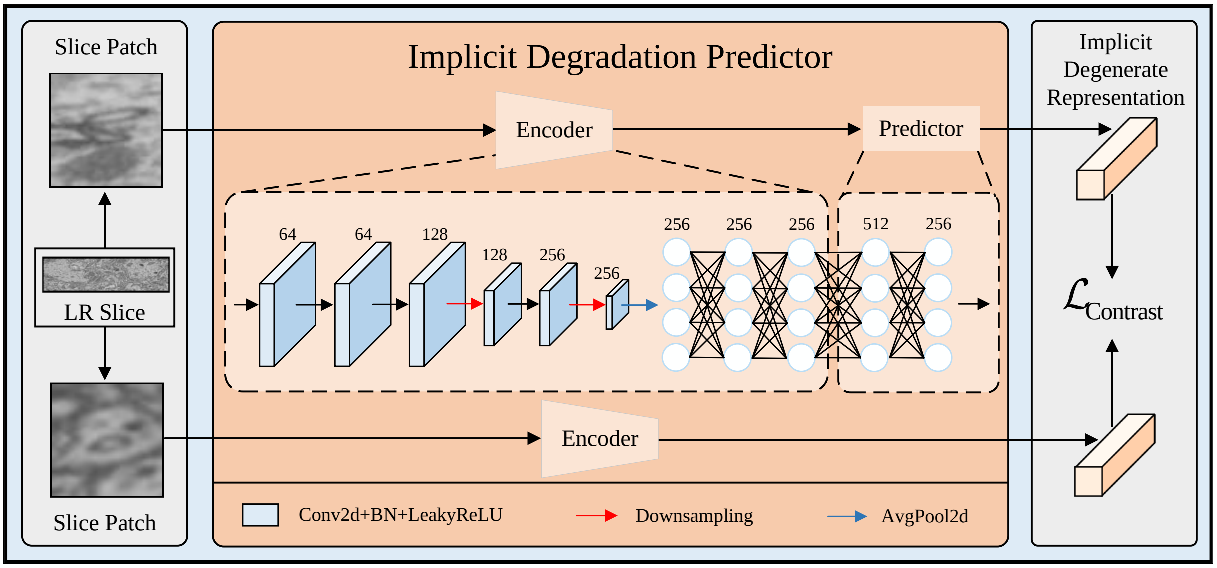

Figure S9: The network structure of implicit degradation predictor for predicting degradation function of low-resolution images.

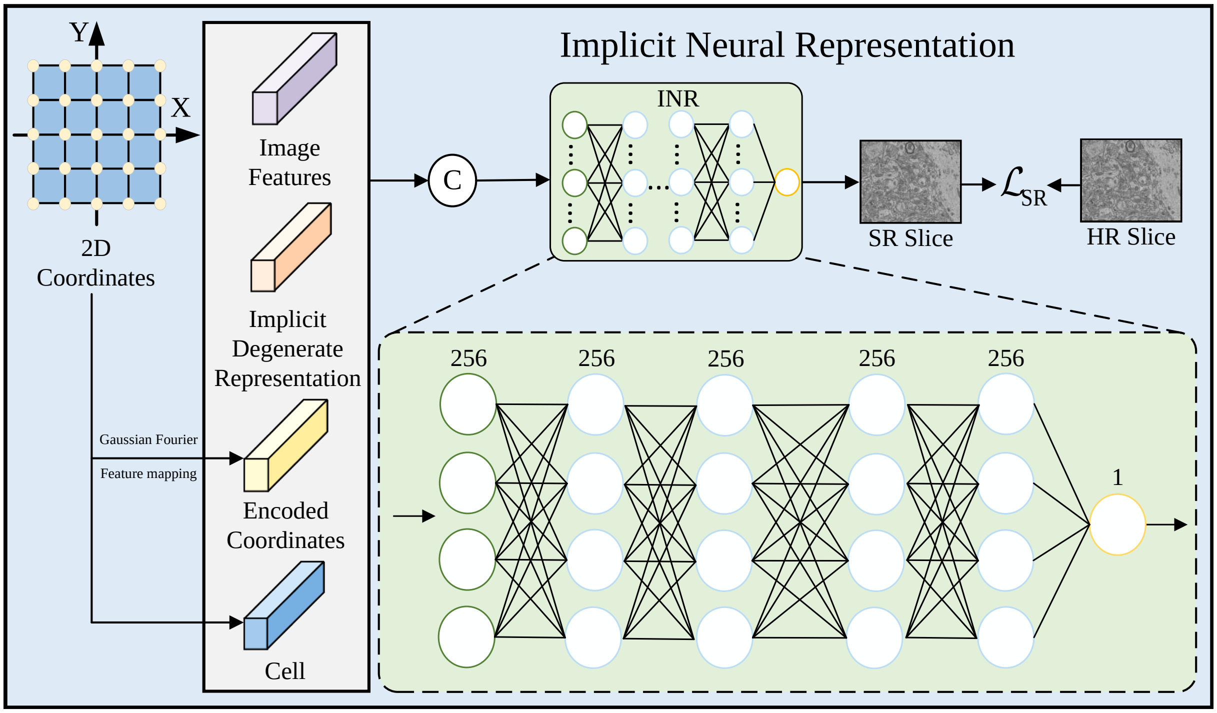

Figure S10: The network structure of implicit neural representation module for upsampling .

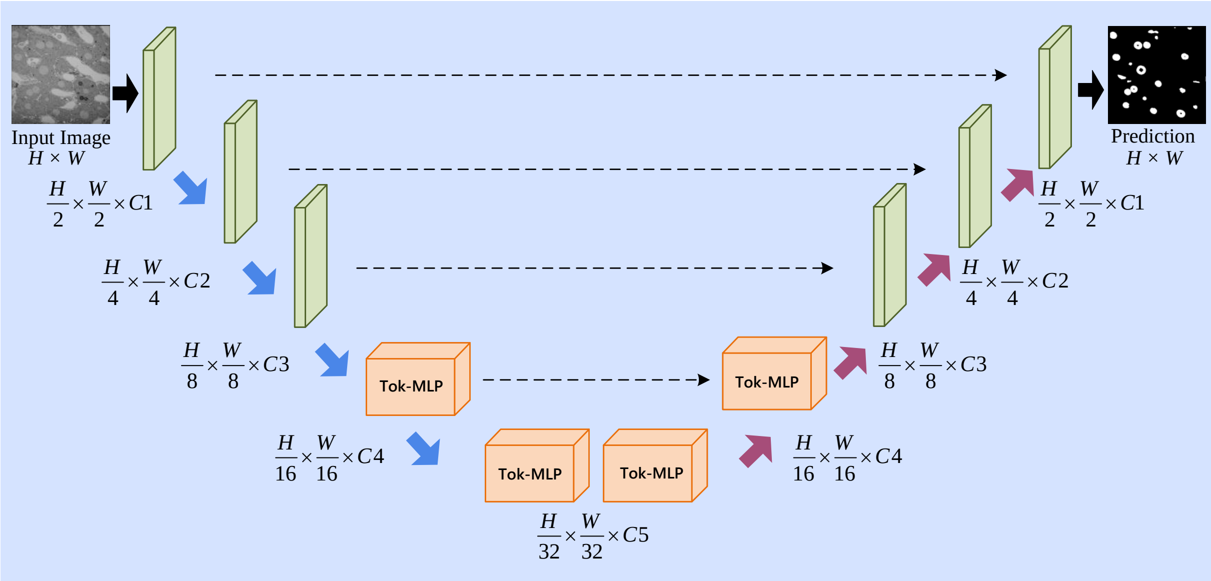

Figure S11: The network structure of the segmentation model used in 3D segmentation.

5 Data Availability

The liver dataset is available at https://openorganelle.janelia.org/datasets/jrc_mus-liver.

The skin dataset can be found at https://openorganelle.janelia.org/datasets/jrc_mus-skin-1.

The kidney dataset is available at https://openorganelle.janelia.org/datasets/jrc_mus-kidney.

The liver-3 dataset can be accessed at https://openorganelle.janelia.org/datasets/jrc_mus-liver-3.

The pancreas dataset is located at https://openorganelle.janelia.org/datasets/jrc_mus-pancreas-4.

The heart dataset can be found at https://openorganelle.janelia.org/datasets/jrc_mus-heart-1.

The EPFL dataset is downloaded from the EPFL website https://www.epfl.ch/labs/cvlab/data/data-em/.

Three Cremi datasets are obtained from the Cremi repository https://Cremi.org/data/.

The Kasthuri11 dataset is located at https://neurodata.io/data/kasthuri15/.

The raw data of neuron segmentation can be found at https://drive.google.com/drive/folders/1OLRxGXOLqDkap8S3cryr2wNC7-_zm-G7?usp=drive_link.

References

[1] C Shan Xu, Song Pang, Gleb Shtengel, Andreas Müller, Alex T Ritter, Huxley K Hoffman, Shin-ya Takemura, Zhiyuan Lu, H Amalia Pasolli, Nirmala Iyer, et al. An open-access volume electron microscopy atlas of whole cells and tissues. *Nature*, 599(7883):147–151, 2021.

[2] Aurélien Lucchi, Yunpeng Li, and Pascal Fua. Learning for structured prediction using approximate subgradient descent with working sets. In *Proceedings of the IEEE Conference on Computer Vision and Pattern Recognition*, pages 1987–1994, 2013.

[3] Yulun Zhang, Yapeng Tian, Yu Kong, Bineng Zhong, and Yun Fu. Residual dense network for image super-resolution. In *Proceedings of the IEEE conference on computer vision and pattern recognition*, pages 2472–2481, 2018.

[4] Xinlei Chen and Kaiming He. Exploring simple siamese representation learning. In *Proceedings of the IEEE/CVF conference on computer vision and pattern recognition*, pages 15750–15758, 2021.

[5] Yinbo Chen, Sifei Liu, and Xiaolong Wang. Learning continuous image representation with local implicit image function. In *Proceedings of the IEEE/CVF conference on computer vision and pattern recognition*, pages 8628–8638, 2021.

[6] Jeya Maria Jose Valanarasu and Vishal M Patel. Unext: Mlp-based rapid medical image segmentation network. In *International conference on medical image computing and computer-assisted intervention*, pages 23–33. Springer, 2022.

[7] Chixiang Lu, Kai Chen, Heng Qiu, Xiaojun Chen, Gu Chen, Xiaojuan Qi, and Haibo Jiang. Diffusion-based deep learning method for augmenting ultrastructural imaging and volume electron microscopy. *Nature Communications*, 15(1):4677, 2024.
